# Functional Analysis of the NucS/EndoMS of the Hyperthermophilic Archaeon *Sulfolobus islandicus* REY15A

**DOI:** 10.3389/fmicb.2020.607431

**Published:** 2020-12-01

**Authors:** Sohail Ahmad, Qihong Huang, Jinfeng Ni, Yuanxi Xiao, Yunfeng Yang, Yulong Shen

**Affiliations:** CRISPR and Archaea Biology Research Center, State Key Laboratory of Microbial Technology, Microbial Technology Institute, Shandong University, Qingdao, China

**Keywords:** archaea, crenarchaea, DNA mismatch repair, Sulfolobus, EndoMS

## Abstract

EndoMS is a recently identified mismatch specific endonuclease in Thermococcales of Archaea and Mycobacteria of Bacteria. The homologs of EndoMS are conserved in Archaea and Actinobacteria, where classic MutS-MutL-mediated DNA mismatch repair pathway is absent or non-functional. Here, we report a study on the *in vitro* mismatch cleavage activity and *in vivo* function of an EndoMS homolog (SisEndoMS) from *Sulfolobus islandicus* REY15A, the model archaeon belonging to Crenarchaeota. SisEndoMS is highly active on duplex DNA containing G/T, G/G, and T/T mismatches. Interestingly, the cleavage activity of SisEndoMS is stimulated by the heterotrimeric PCNAs, and when Mn^2+^ was used as the co-factor instead of Mg^2+^, SisEndoMS was also active on DNA substrates containing C/T or A/G mismatches, suggesting that the endonuclease activity can be regulated by ion co-factors and accessory proteins. We compared the spontaneous mutation rate of the wild type strain REY15A and ∆*endoMS* by counter selection against 5-fluoroorotic acid (5-FOA). The *endoMS* knockout mutant had much higher spontaneous mutation rate (5.06 × 10^−3^) than that of the wild type (4.6 × 10^−6^). A mutation accumulation analysis also showed that the deletion mutant had a higher mutation occurrence than the wild type, with transition mutation being the dominant, suggesting that SisEndoMS is responsible for mutation avoidance in this hyperthermophilic archaeon. Overexpression of the wild type SisEndoMS in *S. islandicus* resulted in retarded growth and abnormal cell morphology, similar to strains overexpressing Hje and Hjc, the Holliday junction endonucleases. Transcriptomic analysis revealed that SisEndoMS overexpression led to upregulation of distinct gene including the CRISPR-Cas IIIB system, methyltransferases, and glycosyltransferases, which are mainly localized to specific regions in the chromosome. Collectively, our results support that EndoMS proteins represent a noncanonical DNA repair pathway in Archaea. The mechanism of the mismatch repair pathway in *Sulfolobus* which have a single chromosome is discussed.

## Introduction

In organisms of all the three domains of life, bacteria, archaea, and eukarya, DNA mismatches frequently occur by replication errors that result in the incorporation of incorrect deoxynucleotides and by other processes such as formation of a heteroduplex between two homologous DNA during recombination, deamination of 5-methylcytosine leading to G:T mismatch, and pair anomalies (Friedberg et al., 2006). The mismatched bases can be repaired by MutS-MutL-based mismatch repair system (canonical MMR) in most Bacteria and Eukarya. MutS and MutL are the key players in the canonical MMR, which are responsible for mismatched base recognition and subsequent strand displacement of the mismatched site for the synthesis of a new DNA strand (Iyer et al., 2006; Kunkel and Erie, 2015). Recently, a mismatch repair specific protein, EndoMS, has been discovered in hyperthermophilic Archaea, *Pyrococcus furiosus* and *Thermococcus kodakarensis*, and Actinobacteria, *Mycobacterium smegmatis* and *Corynebacterium glutamicum* (Ishino et al., 2016; Castaneda-Garcia et al., 2017). The enzyme is a type II restriction-like endonuclease that specifically cleaves both double-stranded DNA strands into 5'-protruding forms, with the mismatched base pair in the central position (Ishino et al., 2016; Nakae et al., 2016). EndoMS protein preferentially cleaves DNA substrates containing G/T, G/G, and T/T mismatches and the activity is enhanced by PCNA or stimulated by the beta clamp (Ishino et al., 2016; Takemoto et al., 2018). Genetic analysis showed that the EndoMS proteins are responsible for the avoidance of spontaneous mutation in Actinobacteria *Mycobacterium smegmatis* and *Corynebacterium glutamicum* (Castaneda-Garcia et al., 2017; Ishino et al., 2018). Because Actinobacteria and most Archaea lack classic MutS-MutL-based MMR system, the discovery of EndoMS could have solved a long-standing mystery of how the mismatches are repaired in these organisms. Questions remain about whether the homologs in other archaeal and bacterial species function similarly in the enzymatic activity, what impact is if the protein is inactivated or upregulated and more importantly, how the DNA double-stranded breaks (DSBs) generated by the enzyme are subsequently processed.

In this study, we investigated the biochemical activity and *in vivo* function of an EndoMS homolog from the genetically tractable model archaeon *Sulfolobus islandicus* REY15A, which belongs to the Crenarchaeota of TACK superphyla (Guy and Ettema, 2011). Our results showed that the enzymatic activity of SisEndoMS resembles that of TkoEndoMS and the deletion of the gene coding for EndoMS led to an elevation of the spontaneous mutation rate. We constructed the strains overexpressing wild type EndoMS and catalysis and DNA binding deficient mutant and compared the difference between strains in growth, cell morphology, and transcriptome. Transcriptomic analysis revealed that the SisEndoMS overexpression led to upregulation of distinct sets of genes, including the CRISPR-Cas IIIB system, methyltransferases, and glycosyltransferases. Our results support that the EndoMS protein represents a noncanonical DNA repair pathway in archaea and bacteria and may help to illuminate the EndoMS-mediated mismatch repair mechanism in prokaryotes.

## Materials and Methods

### Strains and Plasmids

*Sulfolobus islandicus* REY15A (wild type) and *S. islandicus* REY15A (E233S; *pyrEF*-*lacS* double mutant; Deng et al., 2009) were grown in MTSV medium [mineral salt supplemented with 0.2% w/v (weight/volume) tryptone (T), 0.2% (w/v) sucrose (S), mixed solution of vitamins (V)] and MTSVU medium [MTSV supplemented with 0.001% (w/v) uracil (U)], respectively at 75°C as designated previously (Deng et al., 2009). Uracil prototrophic strains were cultivated and selected in MSV medium supplemented with 0.2% casamino acid (C). For protein overexpression, 0.2% arabinose (A) was added in MTV medium. Media plates were solidified with phytagel (0.8%, w/v). Luria-Bertani (LB) medium supplemented with ampicillin (100 μg/ml) was used to grow the *E. coli* DH5α strain harboring the plasmids for gene cloning, whereas LB medium supplemented with chloramphenicol (24 μg/ml) and ampicillin (100 μg/ml) was used to grow the *E. coli* BL21 (DE3) CodonPlus-RIL strains harboring the corresponding plasmids for protein overexpression. All the strains and plasmids used in this study are listed in Supplementary Table S1.

pET15bm, pET22b, or pET30a were used to construct plasmids for the expression of proteins in *E. coli*. pSeSD-based vectors were used for protein expression in *S. islandicus*. The pET vectors were transformed into *E. coli* BL21 (DE3) CodonPlus-RIL or *E. coli* BL21 C43(DE3) pLysS, generating over expression strains of *E. coli*. The pSeSD vectors were transformed into *S. islandicus* REY15A (E233S) by electroporation (Deng et al., 2009), generating strains for the expression in *Sulfolobus*. The allelic replacement method (Zhang et al., 2010) was used to construct the *endoMS* knockout strain. Primers are listed in Supplementary Table S2 and the constructed plasmids and strains were listed in Supplementary Table S1. The details for the construction of the plasmids are available upon request.

### Protein Purification

*E. coli* cells harboring pET15bm-SisEndoMS-N-His plasmid were cultured in LB medium supplemented with ampicillin (100 μg/ml) and chloramphenicol (34 μg/ml) at 37°C with shaking. Isopropyl 1-thio-β-D-galactopyranoside (IPTG) was added to the cells, when the culture OD_600_ reached around 0.4 and the cells were then cultured for 14 h at 16°C. The cells were harvested by centrifugation (7,000 × *g*) for 10 min and resuspended in lysis buffer A (50 mM Tris-HCl, pH 7.0, 500 mM NaCl, and 5% glycerol). Cells were disrupted by sonication followed by centrifugation at 10,000 × *g* for 20 min. The supernatant was incubated at 60°C for 30 min followed by centrifugation (10,000 × *g*, 20 min). The samples were filtered with a 0.45 μm membrane filter. The protein sample was then loaded onto a Ni-NTA agarose column pre-equilibrated in buffer A. Buffer A containing 400 mM imidazole was used as an elute buffer. The protein was subsequently purified with a HiTrap Heparin HP column and eluted by a gradient of buffer B (50 mM Tris-HCl, pH 7.0, 1.5 M NaCl, and 5% glycerol), followed by gel filtration using a Superdex™ 200 10/300 column (GE Healthcare, UK) with buffer A. Similar procedures were used for the purification of SacEndoMS and TgaEndoMS. For purification of PCNAs, *E. coli* cells harboring pET22b plasmids for PCNA subunits (pET22b-SiRe_1594, pET22b-SiRe_1048, and pET22b-SiRe_1602) were cultured in the LB medium supplemented with ampicillin (100 μg/ml) and chloramphenicol (34 μg/ml) at 37°C with shaking. IPTG was added to the cells when OD_600_ ~ 0.4 and cultured at 16°C for 14 h. The cells were harvested, disrupted and resuspended by the same procedure as described above. The proteins were subsequently purified with the HiTrap Heparin HP column (eluted by a gradient of 50 mM Tris-HCl, pH 8.0, 1.5 M NaCl, and 5% glycerol) and by gel filtration using the Superdex™ 200 10/300 column (GE Healthcare, United Kingdom) in buffer A (50 mM Tris-HCl, pH 8.0, 100 mM NaCl, and 5% glycerol). The purified proteins were analyzed by 15% SDS-PAGE.

For the expression and purification of SisEndoMS and catalytically deficient mutants in *Sulfolobus* cells, 1 L cells harboring the corresponding plasmids (Supplementary Table S1) were cultivated in MCSV medium to OD_600_ ~ 0.2. Protein expression was induced by the addition of arabinose (0.2%, w/v) and cultivation at 75°C for 12 h. The cells were resuspended in buffer A (50 mM Tris-HCl, pH 8.0, 300 mM NaCl, and 5% glycerol) after centrifugation (7,000 × *g*) for 10 min. The cells were disrupted by sonication followed by centrifugation (10,000 × *g*) for 20 min. The proteins were filtered with a 0.22 μm membrane filter and loaded onto a Ni-NTA agarose column pre-equilibrated with buffer A. The non-specifically bound proteins were removed by washing with buffer A containing 40 mM imidazole. The bound proteins were eluted afterwards with buffer A containing 400 mM imidazole. Proteins were further purified by gel filtration using the Superdex™ 200 10/300 column (GE Healthcare, United Kingdom) in buffer A.

### Assay for the Mismatch DNA Cleavage Activity of EndoMS

Fluorescently labeled (FAM or Cy5) oligonucleotides were purchased from Sangon Biotech Co., Ltd. (Shanghai, China). Fluorescently labeled substrates (45 bp) having the designed mismatches were made by annealing of the 5'- labeled oligonucleotides (100 μM) with the corresponding complementary oligonucleotides (45 nt and 100 μM) in a buffer containing 7 mM Tris HCl pH 8.0, 50 mM NaCl, and 7 mM MgCl_2_ by heating at 95°C for 10 min and cooling down at room temperature. The mismatch DNA cleavage activity of EndoMS proteins was assayed in a reaction mixture containing DNA substrate (16.7 nM), various concentrations of the enzyme (0 μM, 0.1 μM, and 0.2 μM), 20 mM Tris-HCl (pH 8.0), 6 mM (NH_4_)_2_SO_4_, 100 mM NaCl, 2 mM MgCl_2_/MnCl_2_, and 0.1 mg/ml bovine serum albumin (BSA). The mixtures were incubated at 55°C for 30 min and the reactions were stopped by the addition of 25 mM EDTA and 0.3% SDS. For assay of the activity of TgaEndoMS, the reaction was performed at 80°C for 30 min. The cleavage products were analyzed by 12% PAGE containing 8 M urea. Imaging of cleavage products was carried out with a Typhoon™ FLA 7000 (GE Healthcare) image analyzer.

### Estimation of Mutation Rate and Mutation Profile

*Sulfolobus islandicus* REY15A (wild type) and ∆*endoMS* (EndoMS knockout) strains were grown at 75°C in MTSV and MCSV media. Cultures in the early log phase (nearly OD_600_ 0.2) were taken and diluted in 10^1^, 10^2^, 10^3^, 10^4^, 10^5^, and 10^6^ folds. The cells were spread on MTSV/MCSV plates with or without 5-fluoroorotic acid (5-FOA, 100 μg/ml). The mutation rates were calculated by the number of colonies on the media containing 5-FOA divided by the number of colonies on the media without 5-FOA (Redder and Garrett, 2006; Berkner and Lipps, 2008). The values were averaged based on three independent experiments.

To determine the mutation profile, *S. islandicus* REY15A and ∆*endoMS* were cultured in MTSV media at 75°C, and the culture in early log phase (OD_600_ 0.2) were taken and diluted (10^1^ and 10^2^), and plated on MTSV plates. Single colonies from each strain were isolated and grown in MTSV media. The genomic DNA of *S. islandicus* REY15A and ∆*endoMS* was extracted and sequenced using an Illumina HiSeq 4,000 system (Illumina, San Diego, CA, United States) at the Beijing Genomics Institute (Shenzhen, China). The genomic DNA was trimmed randomly for the construction of read libraries. The paired end fragment libraries were sequenced. Low quality raw reads from the paired end sequencing were discarded. SOAP *de novo* (Ver. 1.05) software was used for the assembling of sequenced reads. Gene prediction of *S. islandicus* REY15A and ∆*endoMS* strains were performed by Glimmer3 software with Hidden Markov models. The average sequencing depth is about 250 times per site. To get the base pair substitutions (BPSs) or indels, the wild type and ∆*endoMS* genome sequences were compared with the reference genome.

### Growth Curves

To acquire strain growth curves, *S. islandicus* cells were grown to early log-phase and the process was repeated three times. Then the cultures were diluted into the medium with an estimated initial OD_600_ value of 0.03. ODs for each strain were monitored every 6 or 12 h. The growth curves were drawn based on data from at least three biological repeats.

### Flow Cytometry

Flow cytometry analysis of the SisEndoMS overexpression strains was performed on Amnis® Imaging flow cytometry (ImageStream®X, MarkII, Merk Millipore, Massachusetts, United States). Cells (0.3 ml) were harvested at early log phase (OD_600_ 0.2) and fixed with 0.7 ml ethanol (70% v/v) for at least 6 h at 4°C, so that the intracellular DNA was able to be stained with propidium iodide (PI). The samples were centrifuged at 800 × *g* for 20 min at 4°C. The supernatant was discarded and the pellets were resuspended in 1 ml phosphate-buffered saline (PBS; 137 mM NaCl, 2.7 mM KCl, 10 mM Na_2_HPO_4_, and 2 mM KH_2_PO_4_, pH 7.4). The samples were centrifuged (800 × *g*, 4°C) again for 20 min and the cells were resuspended in 150 μl PBS buffer containing 50 μg/ml propidium iodide (PI). The stained cells were kept on ice for 1 h. Afterward the samples were analyzed on the Amnis® Imaging Flow Cytometry. The data for each sample were analyzed by IDEAS software (ver. 6.0).

### Scanning Electron Microscopy

For Scanning Electron Microscopy (SEM) using microscope Quanta™ 250 FEG, FEI (Hillsboro, Oregon, United States), the samples were collected at different ODs and centrifuged (800 × *g*) for 5 min. The supernatant was discarded and pellets were resuspended gently with 1 ml PBS (pH 7.4) buffer. Cells were washed (3×) with PBS buffer followed by fixation with 2.5% glutaraldehyde at 4°C for 12 h. After fixation, the samples were dehydrated with a gradient of 30, 50, 70, and 90% (v/v) ethanol for 20 min each. Finally, the samples were treated with absolute ethanol (3×) for around 15 min and centrifuged (800 × *g*, 5 min) and the cells were resuspended with 100 μl absolute ethanol. Samples were dried in a LEICA EM CPD300 (Leica Microsystems, Wetzlar and Mannheim, Germany) dryer and loaded on the specimen stubs sputter-coated with gold (Scientific Instruments Inc., Ted Pella, Blvd. Redding, CA, United States). Image processing of all the samples were accomplished with IDEAS® image data software (Dominical et al., 2017).

### Transcriptomic Analysis

The overexpression strains of the wild type SisEndoMS and the catalytic and DNA binding deficient mutant SisEndoMS (R39E/R67E/W72A/K175A) were grown in the MCSV medium. When the cultures reached OD_600_ 0.2, the protein expression was induced by the addition of arabinose (0.2%, w/v). The cultures were continued to grow for 2 h before the cells were collected by centrifugation (7,000 × *g*, 10 min) and subjected to total RNAs extraction with Trizol (Austin, TX, United Sates). The quality of RNA was analyzed by 1% agarose gel. The RNA sequencing of the samples was carried out by Novogene (Beijing, China). Around 3 μg of RNA per sample was used for the generation of sequencing libraries by the NEBNext Ultra Directional RNA library Prep Kit for Illumina [New England Biolabs (NEB), MA, United Sates] and the quality of the library was analyzed in an Agilent Bioanalyzer 2,100 system. Sample clustering was made on a cBot cluster generation system by TruSeq PE cluster Kit (v3, cBot-HS, Illumina), and the library preparations were sequenced in an Illumina Hiseq platform followed by generation of pair-end reads. The alignment of clean reads to the reference genome of *S. islandicus* REY15A (Guo et al., 2011) was done by aligning clean reads to the reference using Bowtie (ver. 2.2.3) software (Langmead and Salzberg, 2012). The read numbers mapped to each gene was counted by HTSeq (ver. 0.6.1), and the subsequent data were assessed by Fragments Per Kilobase of transcript sequence per Million base pairs sequenced (FPKM) analysis (Trapnell et al., 2012) for the quantification of gene expression levels in *S. islandicus* genome. Differential genome expression analysis (for biological replicates) was performed by DESeq R package (ver. 1.18.0; Wang et al., 2010). DESeq R package provides statistical analysis for differential expression determination in digital gene expression data using a negative binomial distribution-based model. Benjamini and Hochberg’s approach was used for the adjustment of resulting value of *p* to control the false discovery rate. The adjusted value of *p* (0.005) and log2 (fold change) of 1 were set as threshold for significantly differential expression.

## Results

### Expression and Purification of Proteins

To get a better understanding of the properties and functions of EndoMS, we performed biochemical and genetic analysis of the EndoMS homolog (SiRe_0025) in *S. islandicus* REY15A. Multiple sequence alignment analysis showed that the EndoMS from *S. islandicus* REY15A (SisEndoMS) possesses conserved residues for the nuclease catalysis, D159, E173 and K175, as well as substrate binding, R39, R67 and W72 (Supplementary Figure S1). Since the SisEndoMS has high similarity with EndoMS homologs from *Thermococcus kodakarensis* (42.62%) and *Pyrococcus abyssi* (45.50%), the structural model of SisEndoMS could be built (Supplementary Figure S2). As PabNucS and TkoEndoMS, SisEndoMS forms a dimer comprising the N-terminal substrate binding and dimerization domain and the C-terminal catalytic domain (Ren et al., 2009; Nakae et al., 2016).

The wild type and site-directed catalytic deficient mutants D159A, E173A and K175A were expressed in *S. islandicus* REY15A (E233S) with a 6 × His tag at the C-terminal and purified with a nickel column followed by gel filtration as described in the section Materials and Methods (Supplementary Figure S3A). To facilitate the purification of the proteins, the 6 × His tag was added at the N-terminal of SisEndoMS (WT), SisEndoMS-ΔPIP, and SacEndoMS/NucS (EndoMS/NucS from *S. acidocaldarius*) using the pET15bm expression vector. The proteins were purified with Ni-NTA resin, heparin column, followed by gel filtration as described in the section Materials and Methods (Supplementary Figures S3B,C). For the assessment of the effect of PCNA on the endonuclease activity, PCNA subunits were also expressed and purified using pET22b-based vectors (Supplementary Figure S4). In order to get the heterotrimeric PCNA complex, the mixture of three PCNA subunits passed through a gel filtration column. As expected, the heterotrimer complex eluted earlier (around 13 ml) than the individual PCNA subunits (Supplementary Figure S4A). The *Thermococcus gammatolerans* wild type EndoMS/NucS (TgaEndoMS/NucS) and catalytic deficient mutant (TgaEndoMS/NucS-D163A) proteins were expressed and purified from *E. coli* BL21 C43(DE3) pLysS strain using the pET30a vector (Supplementary Figure S3D).

### EndoMS Homologs From the Hyperthermophilic Archaea *Sulfolobus islandicus* and *Sulfolobus acidocaldarius* Exhibit the Mismatch-Specific Endonuclease Activity

**Figure 1:**
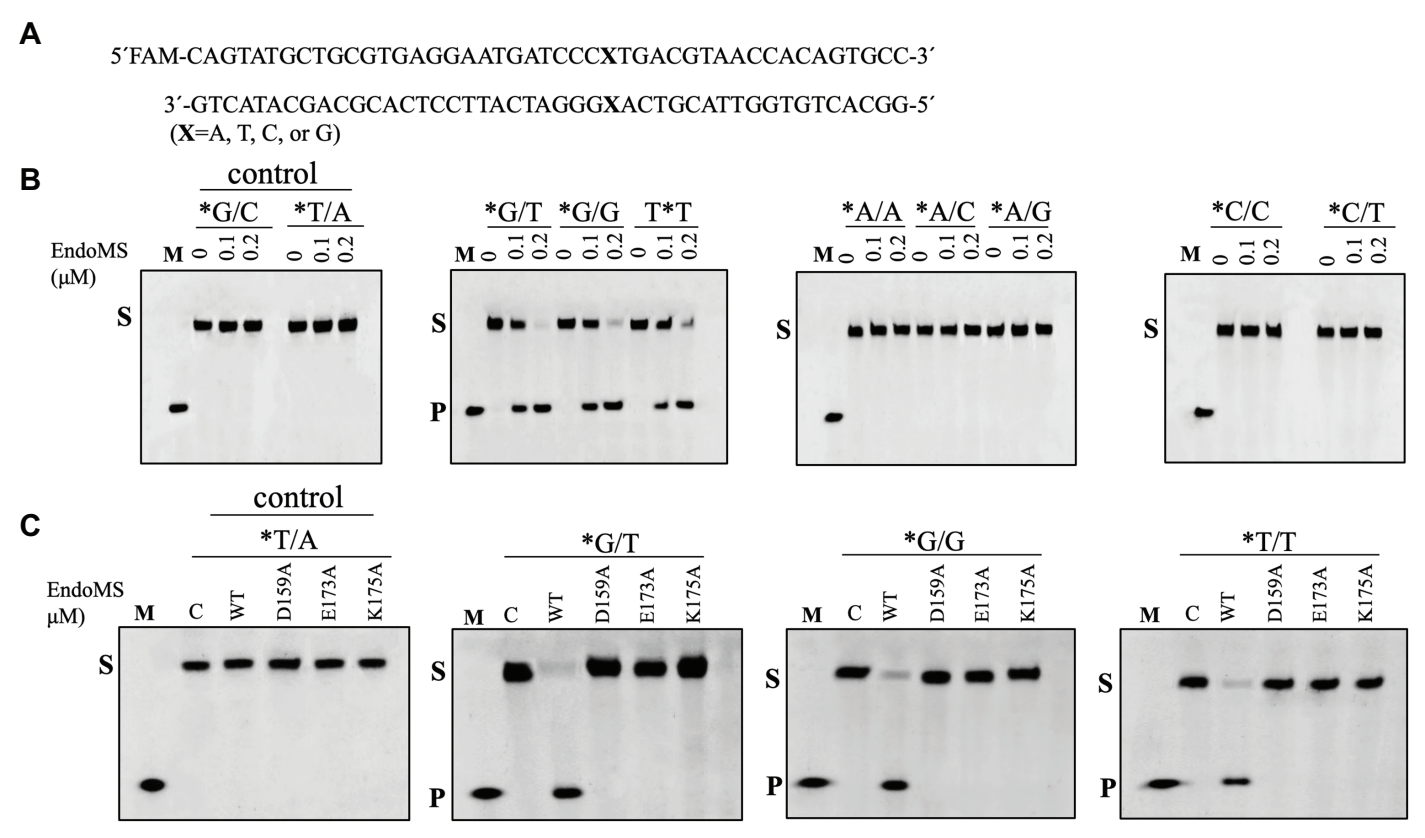
Cleavage of mismatch-containing DNAstrates by SisEndoMS. **(A)** The oligonucleotide duplex substrate (45 bp) containing the mismatched base pairs. The mismatched bases are indicated with boldface letters “X.” The upper strand was labeled with FAM at the 5' end and annealed with the complementary strand to make the duplex substrate. **(B)** The 5'-FAM labeled substrates (0.0167 μM) were incubated with SisEndoMS (WT; 0, 0.1, and 0.2 μM) in a reaction system containing 20 mM Tris-HCl (pH 8.0), 6 mM (NH_4_)_2_SO_4_, 100 mM NaCl, 2 mM MgCl_2_, and 0.1 mg/ml BSA at 55°C for 30 min. **(C)** Assay of the DNA cleavage activity of the catalytic deficient mutants D159A, E173A, and K175A. The reaction conditions were the same as in **(B)** except that only 0.2 μM of the proteins were used. The cleavage products were analyzed by 12% PAGE containing 8 M urea. Imaging of profiles was carried out with a Typhoon™ FLA 7000 image analyzer. C, control without enzyme; S, substrate (45 nt); P, product (24 nt); and M, marker (24 nt). The stars indicate residues within the fluorescently labeled strands.

**Figure 2:**
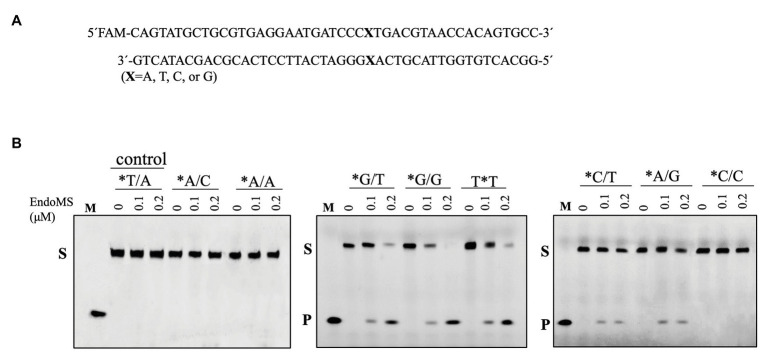
Cleavage of mismatch-containing DNA by SisEndoMS using Mn^2+^ as the co-factor. **(A)** The oligonucleotide duplex substrate (45 bp) containing the mismatched base pairs. The position of the mismatched base pair is indicated with boldface letter “X.” The upper strand was labeled with FAM at the 5' end and annealed with the complementary strand to make the duplex substrate. **(B)** The 5'-FAM labeled substrates (0.0167 μM) were incubated with SisEndoMS (WT; 0, 0.1, and 0.2 μM) in a reaction mixture containing 20 mM Tris-HCl (pH 8.0), 6 mM (NH_4_)_2_SO_4_, 100 mM NaCl, 2 mM MnCl_2_, and 0.1 mg/ml BSA at 55°C for 30 min. The substrates and products were analyzed by 12% PAGE containing 8 M urea. Imaging of the profiles was carried out with a Typhoon™ FLA 7000 image analyzer. S, substrate (45 nt); P, product (24 nt); and M, marker (24 nt). The stars indicate residues within the fluorescently labeled strands.

To assay the mismatch DNA cleavage activity of SisEndoMS, we used fluorescently labeled DNA substrates containing different kinds of mismatched bases. DNA substrates containing G/C and T/A were used as controls. SisEndoMS exhibited cleavage activity against substrates having G/T, G/G and T/T mismatches in the presence of Mg^2+^ as a cofactor (Figure 1). Whereas, it did not exhibit cleavage activity against substrates having A/A, C/C, A/C, T/C, and A/G mismatch bases (Figure 1). The cleavage activity of SisEndoMS against the aforementioned substrates was also analyzed with Mn^2+^ as the cofactor. While SisEndoMS had cleavage activity against substrate containing G/T, G/G, and T/T, it also exhibited weak activity against substrates containing C/T and A/G mismatches in the presence of Mn^2+^ (Figure 2). Since a recent report showing that the *nucS*/*endoMS*-deleted strain of *S. acidocaldarius* (SacEndoMS) did not have high mutation rates, neither it showed any sensitivity to UV irradiation, although it exhibited sensitivity to DNA adducts, therefore, we analyzed the mismatch DNA cleavage activity of SacEndoMS/NucS. The result showed that the SacEndoMS was also able to cleave the DNA substrates having G/T, G/G and T/T mismatches, similar to SisEndoMS (Supplementary Figure S5). We also found that the TgaEndoMS/NucS showed the same cleavage pattern with TkoEndoMS (Supplementary Figure S6).

**Figure 3:**
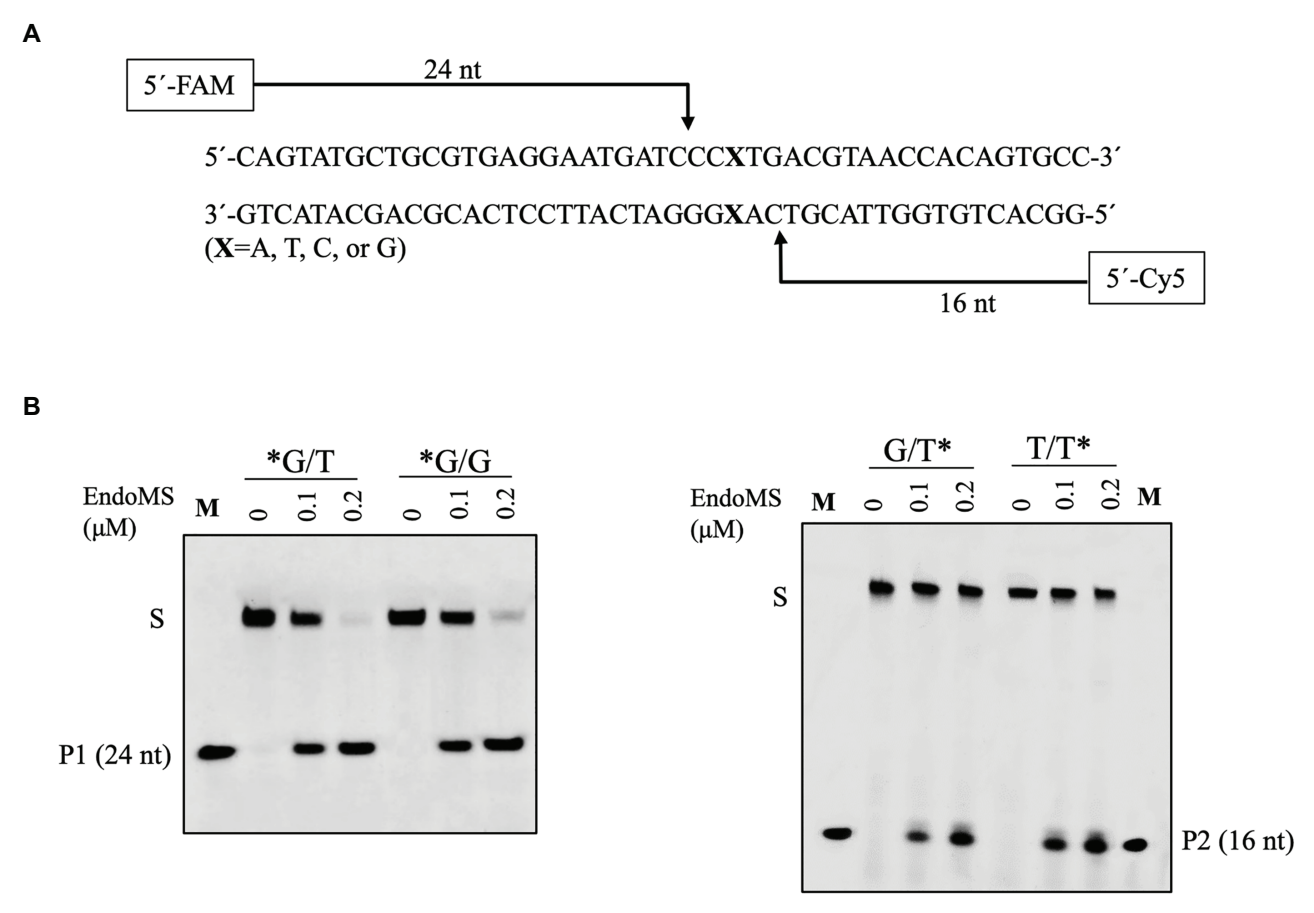
Confirmation of the cleavage pattern of the mismatch-containing DNA by SisEndoMS. **(A)** A schematic map showing the predicted cleavage sites and the labeled products. The upper strand was labeled with FAM and the lower strand with Cy5 at the 5' ends, respectively. **(B)** The 5'FAM/Cy5 labeled DNA substrates (0.0167 μM) were incubated with various concentrations of the SisEndoMS (WT; 0, 0.1, and 0.2 μM) in a reaction mixture as that in Figure 1. The substrates and cleavage products were analyzed by 12% PAGE containing 8 M urea. Imaging of the profiles was carried out with a Typhoon™ FLA 7000 image analyzer. S, substrate; P1 and P2, products, and M, markers (24 nt, left and 16 nt right). The stars indicate residues within the corresponding fluorescence labeled strands.

To confirm the cleavage site of SisEndoMS, we prepared FAM and Cy5 labeled DNA substrates containing mismatched bases at 5' end of upper and lower strands, respectively, and used for the activity assay. As shown in Figure 3, 24 and 16 nt products were generated for the upper strand and lower stand, respectively, suggesting that the SisEndoMS cleaved the third phosphodiester bond on the 5' side of the mismatched base in both strands, leading to the formation of cohesive end with five nucleotides long 5' protrusion, similar to TkoEndoMS.

### The Mismatch DNA Cleavage Activity of SisEndoMS Is Enhanced by Proliferating Cell Nuclear Antigen (PCNA)

**Figure 4:**
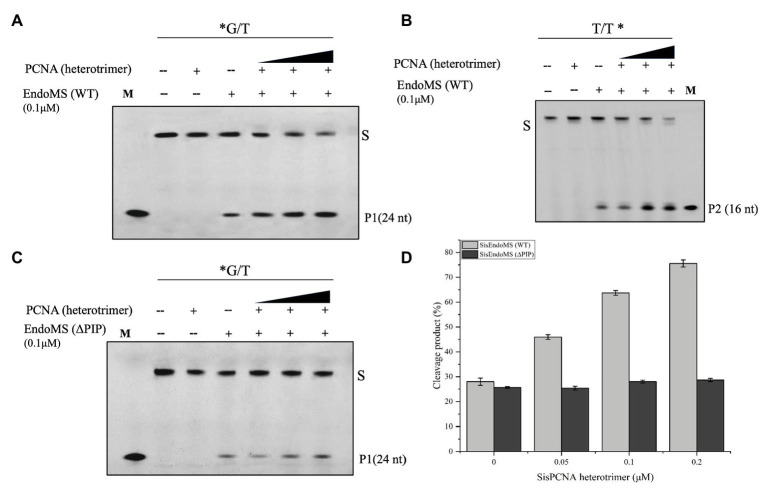
Activation of the mismatch DNA cleavage activity of SisEndoMS by PCNA. The 5'-FAM labeled substrates (*G/T, 0.0167 μM, **A,C**) and 5'-Cy5 labeled substrate (T/T*, 0.0167 μM; **B**) were incubated with SisEndoMS (0.1 μM) and various concentrations of PCNA (as heterotrimer; 0.05, 0.1 and 0.2 μM) in a reaction as that in Figure 1. The substrates and cleavage products were analyzed by 12% denaturing PAGE (8 M urea). Imaging of profiles was carried out with a Typhoon™ FLA 7000 image analyzer. S, substrate (45 nt); P1 and P2, products; and M, marker. **(D)** Quantification of the activity. The results were based on three independent experiments. ImageQuant (ver. 5.2) software was used for the analysis.

In *Sulfolobus* there are three PCNA subunits forming a heterotrimeric ring (Dionne et al., 2003). To investigate whether the PCNAs have any effect on the nuclease activity of SisEndoMS as previously reported in Thermococcales (Ishino et al., 2018), the heterotrimeric PCNA complex was obtained by loading the mixture of purified PCNA subunits SiRe_1048, SiRe_1602, and SiRe_1594 to a gel filtration column and collecting the early elute fractions (Supplementary Figure S4). The putative PIP (PCNA interacting protein) box of SisEndoMS is QKLGLEF, which is located at the C terminus (Supplementary Figure S1). We prepared the truncated SisEndoMS (SisEndoMS-ΔPIP), in which the C-terminal 18 amino acid residues were deleted (Supplementary Figure S3B). The cleavage activity of SisEndoMS-WT was stimulated by increasing concentrations of PCNA (0.05 μM, 0.1 μM, and 0.2 μM, as heterotrimer; Figures 4A,B,D). Whereas, the SisEndoMS-ΔPIP cleavage activity was not enhanced by PCNA (Figures 4C,D).

### EndoMS Is Essential for Mutation Avoidance in *Sulfolobus islandicus*

**Figure 5:**
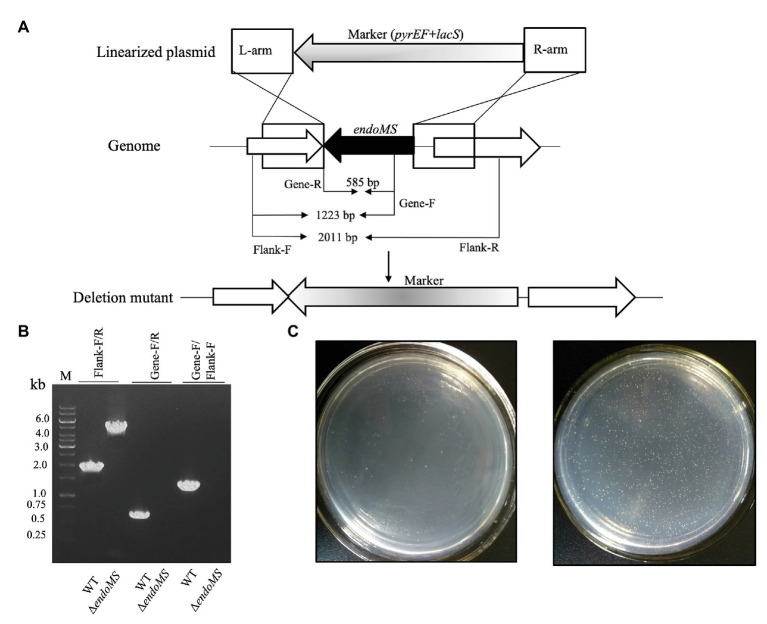
Deletion of *endoMS* resulted in elevated spontaneous mutation rate in *Sulfolobus islandicus*. **(A)** The schematic diagram depicting the allelic replacement strategy used for the construction of the gene knockout strain. The gene coding for EndoMS gene is shown as a black arrow. The marker cassette (*pyrEF* + *lacS*) was inserted into chromosome through double crossover between linearized plasmid and *Sulfolobus islandicus* genome. The primer sites and the lengths of the PCR products were indicated. **(B)** Confirmation of the gene knocks out by PCR. Flanking F/R, gene specific F/R, and Flanking F/Gene F primers were used for PCR verification of the knockout strain. **(C)** Representative plates showing the formation of 5-FOA^R^ colonies of the wild type (left) and *endoMS* knockout (right) strains. Approximately 10^6^ cells (estimated by optical density) were spread on the plates, and the plates were incubated at 75°C for 8 days before counting of the colony numbers.

To investigate the role of SisEndoMS in the maintenance of DNA replication fidelity, we constructed a knockout strain of EndoMS (Δ*endoMS*) of *S. islandicus* using the marker replacement method (Deng et al., 2009; Figures 5A,B). The spontaneous mutation rates of the wild type REY15A and *endoMS* knockout strains were assessed by estimating the mutation frequencies on plates containing 5-FOA (Figure 5C). Notably, a hypermutable phenotype was observed in Δ*endoMS* strain. The mutation rate estimated with 5-FOA resistance was 4.6 × 10^−6^ and 5.06 × 10^−3^ for the wild type and the knockout strains, respectively.

### EndoMS-Mediated Pathway in *Sulfolobus islandicus* Is Specific to Reduce Transition Mutations

In order to analyze the specificity of EndoMS-mediated pathway in *S. islandicus*, the whole genome resequencing analysis of Δ*endoMS* and wild type strains was performed. The mutation occurrence of Δ*endoMS* and wild type strains is summarized in Table 1. The detailed base pair substitutions at different locations of the genome are presented in Supplementary Table S3. There were 88 base pair substitutions in Δ*endoMS*, whereas only eight were identified in the wild type strain. Almost 82.9% (73) of the detected base pair substitutions (88) were biased toward transition mutations for Δ*endoMS*, whereas the remaining 17% (15) were found to be transversion mutations. Among 73 base pair substitutions for Δ*endoMS*, 35 (39.7%) were A:T → G:C and 38 (43.2%) were G:C → A:T types of transition mutations. However, for the wild type, four base pair substitutions were identified as transition (A:T → G:C and G:C → A:T) and four were transversions out of eight totally detected base pair substitutions. The results indicate that the SisEndoMS is responsible for mutation avoidance, predominately transition mutation, typical of mismatch repair deficiency (Castaneda-Garcia et al., 2017).

**Table 1:** Mutational profile by whole genome sequencing.

Mutation types		Type of mismatch	No. of mutations	
			*Sulfolobus islandicus* REY15A	Δ*endoMS*
Transition	A:T → G:C G:C → A:T	A-G	0	10
		T-C	1	25
		G-A	2	17
		C-T	1	21
Transversions	A:T → C:G G:C → T:A A:T → T:A G:C → C:G	A-C	0	1
		T-G	2	2
		G-T	1	1
		A-T	0	6
		T-A	0	3
		G-C	1	1
		C-G	0	1
Overall base pair substitutions		8		88
InDels	Deletion	6		4
	Insertion	13		11

### The Strain Overexpressing SisEndoMS Showed Growth Retardance and Cell Shape Abnormality

**Figure 6:**
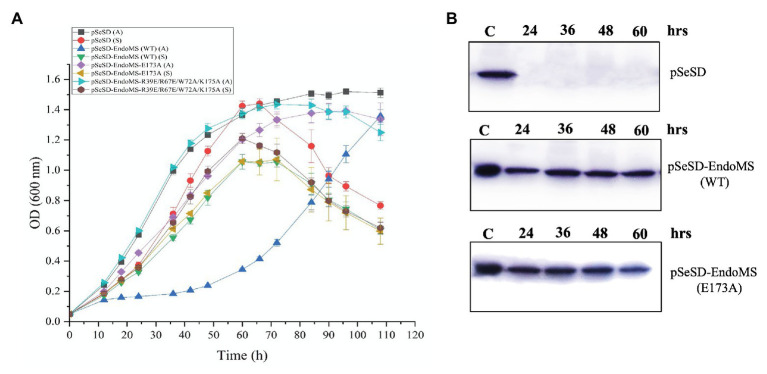
Over-expression of the wild type SisEndoMS greatly inhibited the growth of *Sulfolobus islandicus* REY15A (E233S). **(A)** The growth of E233S harboring the empty vector pSeSD, pSeSD-EndoMS (wild type), pSeSD-EndoMS-E173A (catalytic mutant), and pSeSD-EndoMS-R39E/R67E/W72A/K175A (catalytic and DNA binding mutant) in the presence of sucrose (S) and arabinose (A) was monitored. The growth curves were drawn based on data from at least three biological repeats. **(B)** Western blot analysis for the protein expression of the overexpression strains of pSeSD-EndoMS (WT) and pSeSD-EndoMS (E173A) at different time points. Strain harboring pSeSD (empty vector) was used as a control.

In order to further understand the *in vivo* function of SisEndoMS, we analyzed the effect of overexpression of the wild type, catalysis deficient mutant SisEndoMS (E173A), and DNA binding and catalysis deficient SisEndoMS (R39E/R67E/W72A/E173A) in SisE233S. We assume that the overexpression of the wild type EndoMS would produce double-stranded breaks in the cell and greatly inhibited the cell growth. Arabinose was used as an inducer in this experiment. The growth of SisE233S harboring pSeSD-SisEndoMS, pSeSD-SisEndoMS (E173A), and pSeSD-SisEndoMS (R39E/R67E/W72A/E173A) in MCAV and MCSV was monitored. We noticed a dramatic decrease of the growth of E233S cells harboring pSeSD-EndoMS in the arabinose containing medium (Figure 6A), while the growth of the other two strains did not show much difference comparing with the strain harboring the empty vector. We confirmed that the proteins were overexpressed by Western blot (Figure 6B). The result demonstrated that the nuclease activity of overexpressed wild type EndoMS was detrimental to the cell.

**Figure 7:**
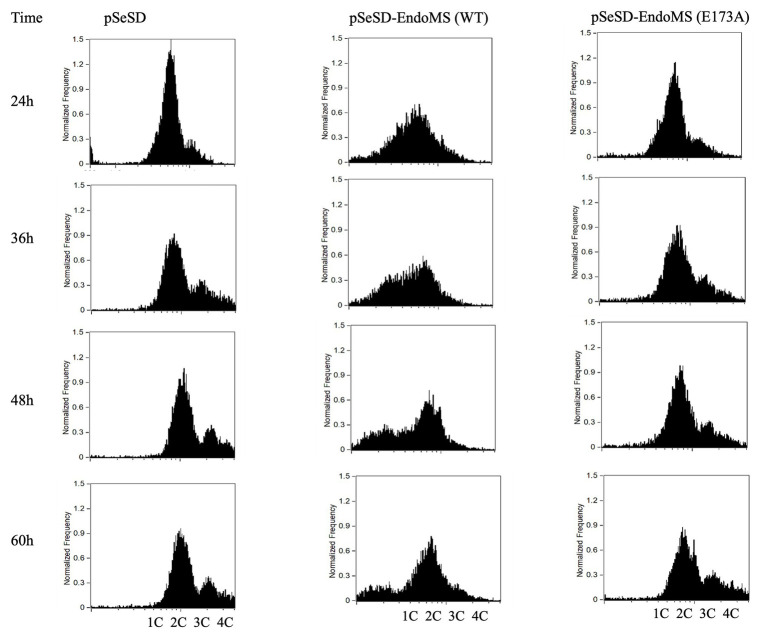
Flow cytometry analysis of the overexpression strains of EndoMS and the catalytic mutants. The strains were cultured at early log phase (OD_600_ 0.2) when the expression of the proteins was induced by the addition of arabinose (0.2% w/v). The samples were taken at different time intervals, fixed with 0.7 ml absolute ethanol (70% v/v) for at least 6 h at 4°C, and analyzed by flow cytometry. The histograms of the strains represent normalized frequency of cells with different DNA content. Strain harboring the empty vector (pSeSD) was used as a control. Cells with one (1C), two (2C), three (3C), and four (4C) copies of chromosomes are indicated at the bottom.

To further validate the effect of the overexpression of SisEndoMS on the growth retardation of E233S, we analyzed the distribution of cells with different DNA content of the stains overexpressing the wild type SisEndoMS and the catalysis deficient mutant EndoMS (E173A) by flow cytometry (Figure 7). Strain overexpressing the wild type SisEndoMS had drastically different DNA profile from those of the strains harboring pSeSD or pSeSD-EndoMS (E173A), with great proportion of DNA less cells at 24, 36, and 48 h. In addition, overexpressing wild-type SisEndoMS appeared to reduce the number of polyploid cells, which would presumably be better at repairing DSBs by HR. The profile for the wild type overexpression strain began to return to normal at 60 h, when the cells start growing, in consistence with the growth curve (Figure 6).

**Figure 8:**
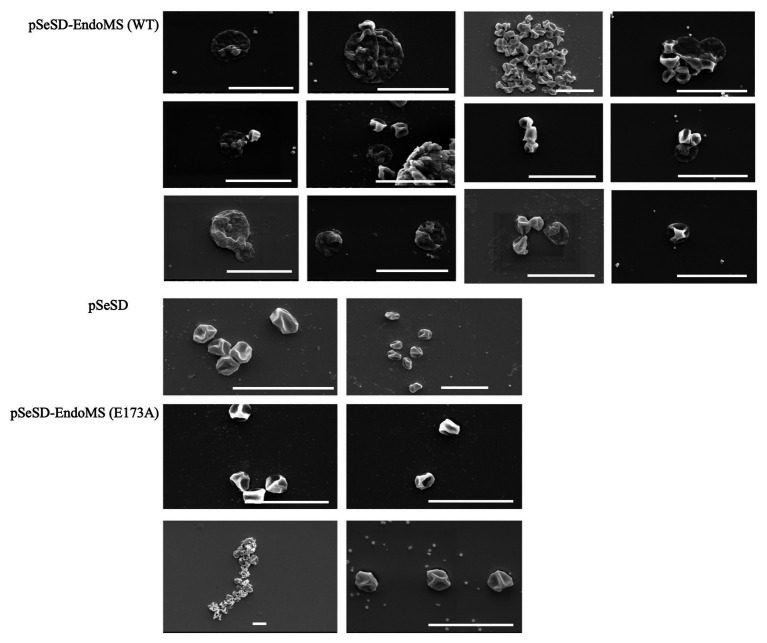
Cell morphology of the strains expressing the wild type EndoMS and the catalytic deficient mutant EndoMS (E173A) by scanning electron microscopy (SEM). The samples were collected after 24 and 36 h of induction and centrifuged at 800 ×g for 5 min. The cells were washed (3×) with PBS buffer (pH 7.4), and fixed with 2.5% glutaraldehyde at 4°C for 12 h and observed under the scanning microscopy. Representative pictures are shown. Scale bars 4 μm.

Scanning Electron Microscopy (SEM) was performed for the overexpression strains and the samples were taken at several time points. Irregular merged cells and enlarged cells were found prominent at 24–48 h. In some cases, normal cells were found apparently attached to the irregular cells in the SisEndoMS overexpression strain (Figure 8). When the culture resumed growing, the cell morphology returned to normal. No significant morphological change was observed in strains overexpressing catalysis deficient mutant and harboring the empty vector.

### Distinct Genes Were Upregulated in the Strain Overexpressing Wild Type SisEndoMS

To get more insight into the *in vivo* function of SisEndoMS and cellular response after the enzymatic activity was executed, we performed comparative transcriptomic analysis of strains overexpressing the wild type and DNA binding and catalytic deficient mutant. Strains harboring pSeSD-SisEndoMS and pSeSD-SisEndoMS (R39E/R67E/W72A/E173A) were cultivated in the MCV medium until OD_600_ reached 0.2. The expression of the proteins was induced by the addition of arabinose, and the samples were taken after induction for 2 h and subjected to transcriptomic analysis. We confirmed that the proteins were expressed after 2 h induction by Western blot analysis (Supplementary Figure S7). Interestingly, comparative transcriptomic analysis revealed that about 60 genes were differentially upregulated and only one gene was downregulated by at least two folds (Supplementary Table S4). There were four distinct features of the regulated genes: (1) the majority of them (49 out of 59) are localized at two chromosomal regions 43 between *sire_0805* and *sire_0897* and six between *sire_0413* and *sire_0418* (Supplementary Table S5); (2) the genes include a CRIPR-Cas IIIB system and several CRISPR-Cas related genes (Supplementary Table S6); (3) many genes involved in modifications including methylation and glycosylation were induced; and (4) some protein transport genes and genes for ROS response, toxin and antitoxin, and transposition were induced.

## Discussion

The EndoMS proteins are conserved in Archaea and Actinobacteria (Ishino et al., 2016; Castaneda-Garcia et al., 2017). The homolog in *Pyrococcus abyssi* was originally identified to be a branched DNA-specific ssDNA nuclease (Ren et al., 2009). The enzyme was later rectified as a mismatch-specific, type II restriction-like endonuclease, which generated double-stranded breaks harboring 5'-protruding with the mismatched base pair in the central position (Ishino et al., 2016; Castaneda-Garcia et al., 2017). So far, the mismatch-specific enzymatic activity of the family enzymes has been reported in *Thermococcus kodakaraensis*, *Pyrococcus furious*, and *Corynebacterium glutamicum*. As for the *in vivo* function, the elevation of spontaneous mutation rate has been observed only in the deletion mutant of *Corynebacterium glutamicum*, with transition mutation being dominant, typical of mismatch repair deficiency (Castaneda-Garcia et al., 2017). In this study, we found that the SisEndoMS exhibited similar mismatch-specific activity to those of the reported EndoMS homolog. In addition, genetic analysis revealed that the SisEndoMS is responsible for mismatch repair. Therefore, our results support that the archaeal EndoMS proteins present a noncanonical mismatch repair pathway.

Interestingly, it was reported that the homolog of *T. gammatolerans* (TgaEndoMS/NucS) had different substrate specificity which generated different cleavage products (Zhang et al., 2020). TgaEndoMS/NucS could cleave uracil (U)- and hypoxanthine (I)-containing dsDNA (U/G and I/T, respectively), and the cleavage sites of the enzyme were at the second phosphodiester on the 5'-site of U or I and at the third phosphodiester on the 5'-site of the opposite base of U or I, creating a double-stranded break with a 4-nt 5'-overhang (Zhang et al., 2020). We found that the TgaEndoMS/NucS showed similar cleavage pattern with SisEndoMS and TkoEndoMS towards DNA substrates having G/T, G/G, and T/T mismatches (Supplementary Figures S6, S8). Intriguingly, SisEndoMS was able to cleave substrate containing U/G mismatch, similar to TgaEndoMS (Supplementary Figure S8), suggesting that SisEndoMS could provide an alternative pathway for repairing deaminated cytosine and probably also deaminated adenine. In another archaeon, *S. acidocaldarius*, deletion of *endoMS* did not result in higher mutation rate and UV sensitivity, although it rendered the strain sensitive to DNA adducts (Suzuki and Kurosawa, 2019). We found that the enzymatic activity of SacEndoMS/NucS was identical to that of SisEndoMS, which is congruous with high sequence similarity between the two enzymes (Supplementary Figure S1). The reason why SacEndoMS/NucS was not responsible for the mutation avoidance is not known.

We analyzed the growth, cell morphology, DNA content, and transcriptome of the strains overexpressing the wild type EndoMS, catalytic deficient and/or catalytic and DNA binding deficient mutants (Figures 6–8). Previously, Hje, the Holliday junction endonuclease was overexpressed in *S. islandicus* and the cells showed growth retardance, similar to what we observed for SisEndoMS overexpression (Huang et al., 2015). We confirmed that the overexpression of either Hje or Hjc, another Holliday junction resolvase, led to growth retardance (Supplementary Figure S9A) and cell morphology abnormality (Supplementary Figure S9C), resembling the phenotype of SisEndoMS overexpression strain. The phenotype of the wild type EndoMS overexpression strain strongly indicates that the enzyme functions as a nuclease *in vivo*, generating harmful double-stranded DNA breaks. Consistently, light microscopy revealed that the abnormal cells in SisEndoMS, Hje, and Hjc overexpressed strains were prone to form cell aggregation (data not shown), a common phenomenon of DNA damage response in *Sulfolobus*. The SEM observation showed that the normal sized cells merged from large cells or clustered cells (Figure 8, Supplementary Figure S9C). How these normal sized cells containing the mutated plasmid were produced needs further investigation.

Interestingly, 60 h after the induction of the strain harboring pSeSD-EndoMS began to grow again. Plasmid is isolated from the culture grownup and we found that the plasmid contained mutations in the EndoMS gene or its promoter region (Supplementary Table S7). We suppose the following scenario would occur for the growth of the strain: (1) the original cells harboring the wild type SisEndoMS stopped growing when the protein was induced and DSB generated at or outside of the replication fork; (2) some cells died, while others triggered an unknown DSB repair process (presumably homologous recombination) to get the damage repair; (3) at the same time, DSB damage triggered mutation occurrence on the plasmid and more mutations were accumulated, producing plasmid with inactive *endoMS*; and (4) cells with chromosomal DSB being repaired and harboring *endoMS* inactive but *pyrEF* active plasmid would divide and survive. Cells with pre-existing spontaneous mutations before induction will survive but because of the low frequency, it would take much longer time for the cells to grow up (Deng et al., 2009; Hong et al., 2012). Therefore, DNA repair and high frequency mutation triggering should have occurred simultaneously for the growth restart.

We revealed instant transcriptomic changes after induction of SisEndoMS. We cannot get clear clues of these changes for specific DNA repair processes from the data. But rather, these changes may show how cells respond to counteract the activity of SisEndoMS leading to DSB generation. These transcriptomic changes may have been brought about by chromosome organization-related gene regulation, because on the genome, the upregulated genes mainly clustered at several regions: *sire_0413–sire_0418*, *sire_0453–sire_0454*, and *sire_0805*–*sire_0897*, with exception of *sire_0596* (Supplementary Table S5). As in other organisms, *Sulfolobus* chromosomes are organized into functional domains, including the formation of chromatin loops (Peeters et al., 2015) and transcription active and inactive compartments (Takemata and Bell, 2020). Interestingly, flanking the largest region (*sire_0805* to *sire_0897*) are transposon genes. It will be interesting to investigate how the transposition of the gene cluster affects gene expression. We also do not know clearly the physiological function of the upregulation of the genes. The upregulated genes include the CRISPR-Cas IIIB system, methyl transferases, glycosyl transferases, and peptide transporters. Among the upregulated genes were those for the CRISPR-Cas IIIB system or related proteins. This system has the RNA-dependent specific RNA/DNA activity as well as non-specific RNA cleavage activity by Csx1 (Supplementary Table S6). It has been proposed that the DNA repair and CRISPR-Cas system have mutual beneficial relationships (Faure et al., 2019). Further study is needed to explore if and how the activation of the CRISPR-Cas IIIB system is beneficial of double-stranded DNA break repair.

**Figure 9:**
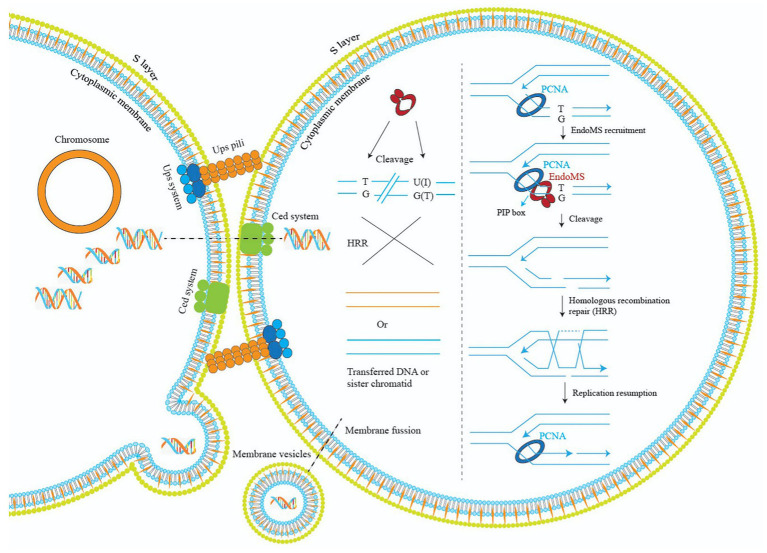
A model illustrating the mechanisms of EndoMS-mediated repair pathway in *Sulfolobus*. Repair of the EndoMS generated DSB at the DNA replication fork requires replication fork stalling and reformation (right). Mismatches (e.g., T/G) and deaminated bases (U/G and I/T) are firstly processed by EndoMS, generating DSBs, which are subsequently repaired by homologous recombination (left). Donor DNAs come from other cells through pili- or membrane vesicle-mediated DNA exchange. The S-layer is omitted for simplicity.

Regarding how the DSB generated by EndoMS is subsequently repaired, for Thermococcales that have multiple copies of chromosomes, it was proposed that the DSB was repaired by homologous recombination. Based on our study, we propose a model to explain the mechanism of EndoMS-mediated mismatch repair in *Sulfolobus* (Figure 9). As a monoploid prokaryotic organism, *Sulfolobus* cells have a long-period of time in the cell cycle containing catenated chromosomes (Lindas and Bernander, 2013). In addition, during DNA replication, there are homologous sister chromatids. The homologous DNA could be utilized by the *Sulfolobus* cell to repair the EndoMS generated DSBs. Repair of the DSB at the DNA replication fork would involve the replication fork stalling and reformation similar to those when cell encounter other DNA lesions (Marians, 2018). Mismatches must also occur outside DNA replication fork, cleavage by SisEndoMS at these mismatches is also processed by homologous recombination, but the donor DNA may come from other cells through pili-mediated DNA exchange (Ajon et al., 2011; van Wolferen et al., 2016). Indeed, we found that overexpression strain exhibited cell clustering, a common phenomenon when cells are treated with DNA damaging agents and pili-mediated DNA exchange occurs. Alternatively, membrane vesicle-mediated gene transfer may be another way to obtain the DNA from other cells (Soler et al., 2008).

## Data Availability Statement

The accession number for the RNA-Seq data in this paper is GEO: GSE158372. The whole genome sequencing data have been deposited at DDBJ/ENA/GenBank under the accessions JADAML000000000 (*Sulfolobus islandicus* REY15A) and JADAMM000000000 (*S. islandicus* REY15A EndoMS Knockout). *S. islandicus* REY15A EndoMS Knockout: BioProject/BioSample identifiers PRJNA664474/SAMN16220128. *S. islandicus* REY15ABioProject/BioSample identifiers PRJNA664470/SAMN1622012.

## Author Contributions

SA, QH, YX, and YY performed the experiments. SA, QH, JN, and YS analyzed the data. SA, QH, and YS designed the experiments and wrote the manuscript. JN help revision of the manuscript. All authors contributed to the article and approved the submitted version.

### Conflict of Interest

The authors declare that the research was conducted in the absence of any commercial or financial relationships that could be construed as a potential conflict of interest.
