# Supplementary material for: Functional Analysis of the NucS/EndoMS of the Hyperthermophilic Archaeon Sulfolobus islandicus REY15A
Source: Front Microbiol. 2020 Dec 1;11:607431. doi: 10.3389/fmicb.2020.607431 (PMC7736090; doi:10.3389/fmicb.2020.607431)
Supplement: Supplementary file 1 [file Data_Sheet_1.docx]

**Table S1.** Plasmids and strains used in the current study.

| Strain/Plasmid | Description | References or sources |
| --- | --- | --- |
| *Sulfolobus islandicus* REY15A | Wild type | Contursi et al. [22] |
| *S. islandicus* REY15A (E233S) | *ΔpyrEFΔlacS* | Deng et al. [23] |
| *Escherichia coli* DH5α | Plasmid amplification | Laboratory strain |
| *E. coli* BL21(DE3) codon plus-RIL | Protein expression | Laboratory strain |
| *E. coli* BL21 C43 (DE3) pLysS | Protein expression | Laboratory strain |
| *E. coli* /pET15bm-SisEndoMS-N-His | Expression of SiRe_0025 having His-tag at the N-terminal | This study |
| *E. coli* /pET15bm-SisEndoMS-ΔPIP-N-His | Expression of SiRe_0025-ΔPIP having His-tag at the N-terminal |  |
| *E. coli* / pET15bm-SacNucS-N-His | Expression of Saci_0200 having His-tag at the N-terminal | This study |
| *E. coli* /pET30a-TgaNucS-C-His | Expression of TgaNucS having His-tag at the C-terminal | This study |
| *E. coli* /pET30a-TgaNucS-D163A-C-His | Expression of TgaNucS catalytic deficient mutant (D163A) having His-tag at the C-terminal | This study |
| ΔSiRe_0025/EndoMS (KO) | *ΔSiRe_0025* | This Study |
| Sis/pSeSD-0025-C-His | E233S harboring pSeSD-SisEndoMS-C-His | This study |
| Sis/pSeSD-0025D159A-C-His | E233S harboring pSeSD-SisEndoMS (D159A)-C-His (catalytic site deficient mutant) | This study |
| Sis/pSeSD-0025E173A-C-His | E233S harboring pSeSD- SisEndoMS (E173A)-C-His (catalytic site deficient mutant) | This study |
| Sis/pSeSD-0025K175A-C-His | E233S harboring pSeSD- SisEndoMS (K175A)-C-His (catalytic site deficient mutant) | This study |
| Sis/pSeSD-SiRe_0025R39E/R67E/W72A-E173A | E233S harboring pSeSD- SisEndoMS (R39E/R67E/W72A-E173A) (DNA binding and catalytic mutant) | This study |
| *E. coli*/pET22b-SiRe_1594 | Expression for the PCNA subunit SiRe_1594 | This study |
| *E. coli*/pET22b-SiRe_1048 | Expression for the PCNA subunit SiRe_1048 | This study |
| *E. coli*/pET22b-SiRe_1602 | Expression for the PCNA subunit SiRe_1602 | This study |

**Table S2:** Oligonucleotides used as primers in the current study.

| Primer | Sequence ^a, b^ (5´-3´) |
| --- | --- |
| pSeSD-SisEndoMS-*Nde*I-F | GGGTTTCATATGGTGTACTCGGTACTACTGAA |
| pSeSD-SisEndoMS-*Sal*I-R | AGCCGTCGACGTAACTAGAATACTTCTGTA |
| pSeSD-SisEndoMS-D159A-F | CTTATGGGAAAATA**GCT**CTGGTGGGGTTAG |
| pSeSD-SisEndoMS-D159A-R | CTAACCCCACCAGAGCTATTTTCCCATAAG |
| pSeSD-SisEndoMS-E173A-F | TTTCGTAATAATT**GCA**GTAAAGAGAAGTAA |
| pSeSD-SisEndoMS-E173A-R | TTACTTCTCTTTACTGCAATTATTACGAAA |
| pSeSD-SisEndoMS-K175A-F | TAATAATTGAAGTA**GCG**AGAAGTAAAGCTC |
| pSeSD-SisEndoMS-K175A-R | GAGCTTTACTTCTCGCTACTTCAATTATTA |
| pET15bm-SisEndoMS-*Nde*I-F | GGGTTTCATATGGTGTACTCGGTACTACTGAA |
| pET15bm-SisEndoMS-*Sal*I-R | GTGCGTCGACTCAGTAACTAGAATACTTCT |
| pET15bm-SisEndoMS-ΔPIP-*Nde*I-F | GGGTTTCATATGGTGTACTCGGTACTACTGAA |
| pET15bm-SisEndoMS-ΔPIP-*Sal*1-R | GACTGTCGACTCATAATAGTTCTCTTGCATG |
| pET15bm-SacNucS-*Nde*1-F | GGTCATATGATGTTCAAGGTATTACTCGAG |
| pET15bm-SacNucS-*Sal*I-R | GGCGGTCGACTTAATCTCTCTGTAAATTTGG |
| pk0025-L-arm-*Sal*I-F | CGGGGTCGACAATTATTCCATGTCCTTTA |
| pk0025-L-arm-*Mlu*I-R | CCGGACGCGTTTACTGAGATAATCGTTGT |
| pk0025-R-arm-*Nco*I-F | CCGCCCATGGAAGAAGGAATAAATTTCCG |
| pk0025-R-arm-*Xho*I-R | CGGCCTCGAGTATAAGATTGCTTAAGGTG |
| pk-0025-flanking-F | GATACCAGTTTTCTTATGGAGTGTGTCAAG |
| pk-0025-specific-F | AAGTAAAGGATGGGGATTTCGTGATTAAAG |
| SiRe_0025R67E/W72A-F | AAGTGTGAAA**GAG**GAACCGTTAAAC**GCA**CAACCACCA |
| SiRe_0025R67E/W72A-R | TGGTGGTTGTGCGTTTAACGGTTCCTCTTTCACACTT |
| SiRe_0025R39E-F | TGAATTATAAGGGA**GAA**GCTGAATCAGTAG |
| SiRe_0025R39E-R | CTACTGATTCAGCTTCTCCCTTATAATTCA |
| SiRe-1594-*Nde*1-F | GGCGGGGTTTCATATGATGATATATCTTA |
| SiRe-1594-*Sal*1-R | GCGCGTCGACTACTATATAGAATTTAGAAA |
| SiRe-1048-*Nde*I-F | GGAATTCCATATGATGAAAGCTAAGGTAATTGA |
| SiRe-1048-*Sal*I-R | ACGCGTCGACTTACTCTGCCCTTGGTGCAA |
| SiRe-1602-*Nde*1-F | GGGCCTCATATGATGTTTAAGATTATTTAC |
| SiRe-1602-*Sal*1-R | ACGCGTCGACTTATAACCTTGGTGCTATCC |

^a^ The underlined denote sites of restriction enzymes. ^b^ The mutated codons are indicated in boldface.

**Table S3.** Mutation sites identified by whole genome sequencing.

| Genome location | Reference | *S. islandicus* REY15A | Δ*endoMS* |
| --- | --- | --- | --- |
| 16674 | T | T | C |
| 232146 | G | G | A |
| 365228 | G | T | T |
| 431555 | G | C | G |
| 431556 | T | G | T |
| 584828 | G | A | A |
| 606967 | T | G | G |
| 606971 | T | C | C |
| 606974 | G | A | A |
| 653519 | G | G | A |
| 728803 | G | G | A |
| 833227 | C | T | T |
| 1232932 | C | C | T |
| 1307694 | C | C | T |
| 1307697 | T | T | C |
| 1307698 | A | A | G |
| 1307700 | T | T | C |
| 1307704 | C | C | T |
| 1307713 | A | A | T |
| 1307730 | T | T | A |
| 1307747 | T | T | C |
| 1307761 | G | G | A |
| 1307766 | C | C | T |
| 1307776 | C | C | T |
| 1307798 | A | A | T |
| 1307816 | A | A | G |
| 1307845 | C | C | T |
| 1307846 | A | A | T |
| 1307856 | T | T | C |
| 1307867 | G | G | A |
| 1307893 | T | T | C |
| 1307894 | T | T | C |
| 1307933 | G | G | A |
| 1307953 | T | T | C |
| 1307957 | A | A | G |
| 1307967 | C | C | T |
| 1307976 | T | T | C |
| 1307986 | C | C | G |
| 1307993 | G | G | A |
| 1307994 | G | G | T |
| 1307995 | A | A | G |
| 1307999 | C | C | T |
| 1308011 | C | C | T |
| 1308017 | C | C | T |
| 1308029 | A | A | T |
| 1308053 | C | C | T |
| 1308067 | T | T | C |
| 1308101 | T | T | G |
| 1308109 | G | G | A |
| 1308115 | G | G | A |
| 1308122 | G | G | A |
| 1308141 | T | T | C |
| 1308164 | T | T | C |
| 1308179 | A | A | G |
| 1308198 | C | C | T |
| 1308213 | T | T | C |
| 1308223 | C | C | T |
| 1308237 | A | A | T |
| 1308266 | G | G | A |
| 1308278 | A | A | C |
| 1308283 | T | T | C |
| 1308288 | T | T | C |
| 1308291 | T | T | A |
| 1308295 | G | G | A |
| 1308313 | G | G | C |
| 1308314 | T | T | C |
| 1308329 | T | T | A |
| 1308332 | C | C | T |
| 1308335 | C | C | A |
| 1308346 | T | T | C |
| 1308374 | T | T | C |
| 1308385 | T | T | C |
| 1308386 | C | C | T |
| 1308462 | T | T | C |
| 1308477 | T | T | C |
| 1308483 | C | C | T |
| 1308498 | T | T | C |
| 1308530 | A | A | G |
| 1308551 | A | A | G |
| 1308599 | A | A | G |
| 1308618 | C | C | T |
| 1308624 | A | A | G |
| 1308663 | A | A | T |
| 1308675 | A | A | G |
| 1308678 | G | G | A |
| 1308687 | T | T | C |
| 1308696 | C | C | T |
| 1308729 | G | G | A |
| 1308738 | G | G | A |
| 1308753 | C | C | T |
| 1308775 | C | C | T |
| 1412165 | T | T | G |
| 1644735 | C | C | C |
| 2069791 | T | T | C |

**Supplementary Table S4.** Summary of genes with >2 folds transcriptional change (ordered according to regulation levels). The data were based on comparative transcriptomic analysis of strains overexpressing the wild type EndoMS and the catalytic deficient mutant.

| **Gene_id** | **Reads_mutant** | **Reads_WT** | **Log2 (WT/mutant)** | **Name** | **Description** |
| --- | --- | --- | --- | --- | --- |
| SiRe_0805 | 1.620785832 | 18.24239068 | 3.4925 | - | Uncharacterized protein |
| SiRe_0880 | 76.16891659 | 515.2703262 | 2.7581 | AglR | MATE family membrane protein, Rfbx family, flippase |
| SiRe_0857 | 3.226802428 | 21.46741894 | 2.734 | - | Transposase |
| SiRe_0807 | 6.239741313 | 37.2651478 | 2.5783 | ArgE | Acetylornithine deacetylase/Succinyl-diaminopimelate desuccinylase or related deacylase |
| SiRe_0878 | 104.6710962 | 619.0910359 | 2.5643 | - | SAM-dependent methyltransferase |
| SiRe_0883 | 15.61307859 | 91.39702855 | 2.5494 | - | ATPase, AAA+ superfamily fused to HTH and PD-(DE)xK endonuclease domains |
| SiRe_0879 | 577.053262 | 3026.613631 | 2.3909 | CysH | 3'-phosphoadenosine 5'-phosphosulfate sulfotransferase (PAPS reductase)/FAD synthetase |
| SiRe_0806 | 161.7264421 | 803.8574619 | 2.3134 | - | PIN domain |
| SiRe_0882 | 13.56915672 | 62.5667397 | 2.2051 |  |  |
| SiRe_0881 | 19.0685138 | 86.13559852 | 2.1754 | - | SAM-dependent methyltransferase |
| SiRe_0868 | 49.812458 | 217.4591072 | 2.1262 | - | Glycosyl transferase family 2 |
| SiRe_0867 | 40.569956 | 173.0337897 | 2.0926 | RfaG | Glycosyltransferase |
| SiRe_0876 | 208.9505957 | 881.1796787 | 2.0763 | - | Glycosyl transferase family 2 |
| SiRe_0416 | 275.7871432 | 1147.805023 | 2.0573 | DppC | ABC-type dipeptide/oligopeptide/nickel transport system, permease component |
| SiRe_0417 | 491.7196184 | 2013.659523 | 2.0339 | DppB | ABC-type dipeptide/oligopeptide/nickel transport system, permease component |
| SiRe_0869 | 177.7692399 | 716.7592898 | 2.0115 | - | Uncharacterized membrane protein |
| SiRe_0824 | 4.942795551 | 19.75619949 | 1.9989 | YcaO | Ribosomal protein S12 methylthiotransferase accessory factor YcaO |
| SiRe_0874 | 9.457114784 | 37.27594182 | 1.9788 | - | Glycosyl transferase family 2 |
| SiRe_0877 | 140.0388338 | 547.3003109 | 1.9665 | LmbE | α-N-acetylglucosaminidase or N-acetylglucosaminyl deacetylase, LmbE family |
| SiRe_0875 | 695.5541808 | 2703.899862 | 1.9588 | - | Arylsulfatase A or related enzyme |
| SiRe_0830 | 8.278322907 | 30.93625518 | 1.9019 | - | Uncharacterized membrane protein |
| SiRe_0841 | 28.87441608 | 105.2980482 | 1.8666 | RfbA | dTDP-glucose pyrophosphorylase |
| SiRe_0854 | 12.86773992 | 45.07123015 | 1.8084 | RfaG | Glycosyltransferase |
| SiRe_0840 | 33.19623288 | 115.6544126 | 1.8007 | RfbB | dTDP-D-glucose 4,6-dehydratase |
| SiRe_0453 | 297.2548799 | 996.9925865 | 1.7459 | - | Protein distantly related to bacterial ferritins |
| SiRe_0418 | 12783.60378 | 42243.07281 | 1.7244 | - | Extracellular solute-binding protein with Ig-fold domain |
| SiRe_0837 | 59.62295133 | 196.963421 | 1.724 | - | ATPase, predicted component of phage defense system |
| SiRe_0809 | 63.70011452 | 208.8151636 | 1.7129 | VapC | PIN domain containing protein |
| SiRe_0454 | 8.497526729 | 27.29579566 | 1.6836 | {NirD} | Ferredoxin subunit of nitrite reductase or ring-hydroxylating dioxygenase |
| SiRe_0413 | 17.72600873 | 56.7914294 | 1.6798 | - | Uncharacterized membrane protein |
| SiRe_0843 | 27.89396928 | 88.46271924 | 1.6651 | RfbC | dTDP-4-dehydrorhamnose 3,5-epimerase or related enzyme |
| SiRe_0127 | 7.512488881 | 23.45486613 | 1.6425 | - | Uncharacterized membrane protein |
| SiRe_0820 | 26.64082886 | 82.23687649 | 1.6261 | - | Uncharacterized protein |
| SiRe_0415 | 338.3728831 | 1041.505407 | 1.622 | DppD | ABC-type dipeptide/oligopeptide/nickel transport system, ATPase component |
| SiRe_0850 | 65.63865091 | 201.300066 | 1.6167 | - | Glycosyl transferase family 2 |
| SiRe_0844 | 8.865674506 | 26.82264632 | 1.5971 | - | SAM-dependent methyltransferase |
| SiRe_0870 | 13.83800853 | 40.51489787 | 1.5498 |  |  |
| SiRe_0818 | 21.98415755 | 61.82355577 | 1.4917 | - | PIN domain containing protein |
| SiRe_0863 | 28.94467595 | 81.09936031 | 1.4864 | - | SAM-dependent methyltransferase |
| SiRe_0864 | 13.62948526 | 38.18826617 | 1.4864 | - | SAM-dependent methyltransferase |
| SiRe_0897 | 31.34197636 | 87.76381464 | 1.4855 | - | ATPase, AAA+ superfamily fused to HTH and PD-(DE)xK endonuclease domains |
| SiRe_0815 | 24.45864358 | 68.11631834 | 1.4777 | - | Uncharacterized protein |
| SiRe_0893 | 46.15309058 | 126.9936439 | 1.4603 | Cmr6g7 | CRISPR-Cas system related protein, RAMP superfamily Cas7 group |
| SiRe_0894 | 197.8364777 | 539.1651032 | 1.4464 | Cas10 | CRISPR associated Cas10, large subunit of Type III effector complex, contains HD family nuclease |
| SiRe_0895 | 66.7700425 | 179.0993027 | 1.4235 | Cmr3g5 | CRISPR-Cas system related protein, RAMP superfamily Cas5 group |
| SiRe_0853 | 202.5733402 | 538.0417334 | 1.4093 | - | SAM-dependent methyltransferase |
| SiRe_0414 | 227.5637577 | 603.4823924 | 1.407 | AppF | ABC-type oligopeptide transport system, ATPase component |
| SiRe_0596 | 622.8235954 | 1651.400903 | 1.4068 | - | HEPN domain containing protein |
| SiRe_0842 | 26.48904782 | 65.96034496 | 1.3162 | RfbD | dTDP-4-dehydrorhamnose reductase |
| SiRe_0838 | 24.34866706 | 59.27939249 | 1.2837 | - | TOPRIM family nucleotidyl transferase/hydrolase domain, DUF3226 family |
| SiRe_0861 | 168.6791898 | 409.4349552 | 1.2794 | - | Glycosyl transferase family 2 |
| SiRe_0884 | 55.41111105 | 129.7260397 | 1.2272 | Csx1 | CARF domain containing protein, contains HTH and HEPN domains |
| SiRe_0845 | 24.2694806 | 55.2250432 | 1.1862 | - | Transposon, IS605 OrfB family, contains RNAse H fold nuclease and Zn finger domains |
| SiRe_0891 | 22.92313372 | 51.75776154 | 1.175 | Cmr5SS | CRISPR-Cas system related protein, small subunit of CASCADE complex |
| SiRe_0811 | 86.27320914 | 187.8249314 | 1.1224 | - | CARF and HTH domains containing protein |
| SiRe_0817 | 61.18415785 | 130.6006689 | 1.0939 | - | HEPN domain containing protein |
| SiRe_0819 | 24.40081824 | 51.02794198 | 1.0644 | - | Uncharacterized protein |
| SiRe_0892 | 134.5993029 | 276.1318002 | 1.0367 | Cmr1g7 | CRISPR-Cas system related protein, RAMP superfamily Cas7 group |
| SiRe_0890 | 111.2995972 | 227.7043705 | 1.0327 | Cmr4g7 | CRISPR-Cas system related protein, RAMP superfamily Cas7 group |
| SiRe_1331 | 584.0643257 | 289.2907664 | -1.0136 | GltB | Glutamate synthase domain 2 and ferredoxin domain |

**Supplementary Table S5.** List of genes with >2 folds transcriptional change (ordered according the gene codes). The data were obtained from comparative transcriptomic analysis of strains overexpressing the wild type EndoMS and the catalytic deficient mutant.

| **Gene_id** | **Reads_mutant** | **Reads_WT** | **Log2 (WT/mutant)** | **Name** | **Description** |
| --- | --- | --- | --- | --- | --- |
| SiRe_0127 | 7.51248888 | 23.4548661 | 1.6425 | - | Uncharacterized membrane protein |
| SiRe_0413 | 17.7260087 | 56.7914294 | 1.6798 | - | Uncharacterized membrane protein |
| SiRe_0414 | 227.563758 | 603.482392 | 1.407 | AppF | ABC-type oligopeptide transport system, ATPase component |
| SiRe_0415 | 338.372883 | 1041.50541 | 1.622 | DppD | ABC-type dipeptide/oligopeptide/nickel transport system, ATPase component |
| SiRe_0416 | 275.787143 | 1147.80502 | 2.0573 | DppC | ABC-type dipeptide/oligopeptide/nickel transport system, permease component |
| SiRe_0417 | 491.719618 | 2013.65952 | 2.0339 | DppB | ABC-type dipeptide/oligopeptide/nickel transport system, permease component |
| SiRe_0418 | 12783.6038 | 42243.0728 | 1.7244 | - | Extracellular solute-binding protein with Ig-fold domain |
| SiRe_0453 | 297.25488 | 996.992587 | 1.7459 | - | Protein distantly related to bacterial ferritins |
| SiRe_0454 | 8.49752673 | 27.2957957 | 1.6836 | {NirD} | Ferredoxin subunit of nitrite reductase or ring-hydroxylating dioxygenase |
| SiRe_0596 | 622.823595 | 1651.4009 | 1.4068 | - | HEPN domain containing protein |
| SiRe_0805 | 1.62078583 | 18.2423907 | 3.4925 | - | Uncharacterized protein |
| SiRe_0806 | 161.726442 | 803.857462 | 2.3134 | - | PIN domain |
| SiRe_0807 | 6.23974131 | 37.2651478 | 2.5783 | ArgE | Acetylornithine deacetylase/Succinyl-diaminopimelate desuccinylase or related deacylase |
| SiRe_0809 | 63.7001145 | 208.815164 | 1.7129 | VapC | PIN domain containing protein |
| SiRe_0811 | 86.2732091 | 187.824931 | 1.1224 | - | CARF and HTH domains containing protein |
| SiRe_0815 | 24.4586436 | 68.1163183 | 1.4777 | - | Uncharacterized protein |
| SiRe_0817 | 61.1841579 | 130.600669 | 1.0939 | - | HEPN domain containing protein |
| SiRe_0818 | 21.9841576 | 61.8235558 | 1.4917 | - | PIN domain containing protein |
| SiRe_0819 | 24.4008182 | 51.027942 | 1.0644 | - | Uncharacterized protein |
| SiRe_0820 | 26.6408289 | 82.2368765 | 1.6261 | - | Uncharacterized protein |
| SiRe_0824 | 4.94279555 | 19.7561995 | 1.9989 | YcaO | Ribosomal protein S12 methylthiotransferase accessory factor YcaO |
| SiRe_0830 | 8.27832291 | 30.9362552 | 1.9019 | - | Uncharacterized membrane protein |
| SiRe_0837 | 59.6229513 | 196.963421 | 1.724 | - | ATPase, predicted component of phage defense system |
| SiRe_0838 | 24.3486671 | 59.2793925 | 1.2837 | - | TOPRIM family nucleotidyl transferase/hydrolase domain, DUF3226 family |
| SiRe_0840 | 33.1962329 | 115.654413 | 1.8007 | RfbB | dTDP-D-glucose 4,6-dehydratase |
| SiRe_0841 | 28.8744161 | 105.298048 | 1.8666 | RfbA | dTDP-glucose pyrophosphorylase |
| SiRe_0842 | 26.4890478 | 65.960345 | 1.3162 | RfbD | dTDP-4-dehydrorhamnose reductase |
| SiRe_0843 | 27.8939693 | 88.4627192 | 1.6651 | RfbC | dTDP-4-dehydrorhamnose 3,5-epimerase or related enzyme |
| SiRe_0844 | 8.86567451 | 26.8226463 | 1.5971 | - | SAM-dependent methyltransferase |
| SiRe_0845 | 24.2694806 | 55.2250432 | 1.1862 | - | Transposon IS605 OrfB family, contains RNAse H fold nuclease and Zn finger domains |
| SiRe_0850 | 65.6386509 | 201.300066 | 1.6167 | - | Glycosyl transferase family 2 |
| SiRe_0853 | 202.57334 | 538.041733 | 1.4093 | - | SAM-dependent methyltransferase |
| SiRe_0854 | 12.8677399 | 45.0712302 | 1.8084 | RfaG | Glycosyltransferase |
| SiRe_0857 | 3.22680243 | 21.4674189 | 2.734 | - | Transposase |
| SiRe_0861 | 168.67919 | 409.434955 | 1.2794 | - | Glycosyl transferase family 2 |
| SiRe_0863 | 28.944676 | 81.0993603 | 1.4864 | - | SAM-dependent methyltransferase |
| SiRe_0864 | 13.6294853 | 38.1882662 | 1.4864 | - | SAM-dependent methyltransferase |
| SiRe_0867 | 40.569956 | 173.03379 | 2.0926 | RfaG | Glycosyltransferase |
| SiRe_0868 | 49.812458 | 217.459107 | 2.1262 | - | Glycosyl transferase family 2 |
| SiRe_0869 | 177.76924 | 716.75929 | 2.0115 | - | Uncharacterized membrane protein |
| SiRe_0870 | 13.8380085 | 40.5148979 | 1.5498 |  |  |
| SiRe_0874 | 9.45711478 | 37.2759418 | 1.9788 | - | Glycosyl transferase family 2 |
| SiRe_0875 | 695.554181 | 2703.89986 | 1.9588 | - | Arylsulfatase A or related enzyme |
| SiRe_0876 | 208.950596 | 881.179679 | 2.0763 | - | Glycosyl transferase family 2 |
| SiRe_0877 | 140.038834 | 547.300311 | 1.9665 | LmbE | α-N-acetylglucosaminidase or N-acetylglucosaminyl deacetylase, LmbE family |
| SiRe_0878 | 104.671096 | 619.091036 | 2.5643 | - | SAM-dependent methyltransferase |
| SiRe_0879 | 577.053262 | 3026.61363 | 2.3909 | CysH | 3'-phosphoadenosine 5'-phosphosulfate sulfotransferase (PAPS reductase)/FAD synthetase |
| SiRe_0880 | 76.1689166 | 515.270326 | 2.7581 | AglR | MATE family membrane protein, Rfbx family, flippase |
| SiRe_0881 | 19.0685138 | 86.1355985 | 2.1754 | - | SAM-dependent methyltransferase |
| SiRe_0882 | 13.5691567 | 62.5667397 | 2.2051 |  |  |
| SiRe_0883 | 15.6130786 | 91.3970286 | 2.5494 | - | ATPase, AAA+ superfamily fused to HTH and PD-(DE)xK endonuclease domains |
| SiRe_0884 | 55.4111111 | 129.72604 | 1.2272 | Csx1 | CARF domain containing protein, contains HTH and HEPN domains |
| SiRe_0890 | 111.299597 | 227.704371 | 1.0327 | Cmr4g7 | CRISPR-Cas system related protein, RAMP superfamily Cas7 group |
| SiRe_0891 | 22.9231337 | 51.7577615 | 1.175 | Cmr5SS | CRISPR-Cas system related protein, small subunit of CASCADE complex |
| SiRe_0892 | 134.599303 | 276.1318 | 1.0367 | Cmr1g7 | CRISPR-Cas system related protein, RAMP superfamily Cas7 group |
| SiRe_0893 | 46.1530906 | 126.993644 | 1.4603 | Cmr6g7 | CRISPR-Cas system related protein, RAMP superfamily Cas7 group |
| SiRe_0894 | 197.836478 | 539.165103 | 1.4464 | Cas10 | CRISPR associated large subunit of Type III effector complex, contains HD family nuclease |
| SiRe_0895 | 66.7700425 | 179.099303 | 1.4235 | Cmr3g5 | CRISPR-Cas system related protein, RAMP superfamily Cas5 group |
| SiRe_0897 | 31.3419764 | 87.7638146 | 1.4855 | - | ATPase, AAA+ superfamily fused to HTH and PD-(DE)xK endonuclease domains |
| SiRe_1331 | 584.064326 | 289.290766 | -1.0136 | GltB | Glutamate synthase domain 2 and ferredoxin domain |

**Supplementary Table S6.** Summary of genes with >2 folds increase in transcriptional level based on comparative transcriptomic analysis of strains overexpressing the wild type EndoMS and the catalytic deficient mutant.

| **Categories** | **Gene_id** | **Name** | **Description** | **Log2 (WT/mutant)** |
| --- | --- | --- | --- | --- |
| CRISPR-Cas or related | SiRe_0893 | Cmr6g7 | CRISPR-Cas system related protein, RAMP superfamily Cas7 group | 1.4603 |
|  | SiRe_0894 | Cas10 | CRISPR associated Cas10, large subunit of Type III effector complex, contains HD family nuclease | 1.4464 |
|  | SiRe_0895 | Cmr3g5 | CRISPR-Cas system related protein, RAMP superfamily Cas5 group | 1.4235 |
|  | SiRe_0884 | Csx1 | CARF domain containing protein, contains HTH and HEPN domains | 1.2272 |
|  | SiRe_0891 | Cmr5SS | CRISPR-Cas system related protein, small subunit of CASCADE complex | 1.175 |
|  | SiRe_0892 | Cmr1g7 | CRISPR-Cas system related protein, RAMP superfamily Cas7 group | 1.0367 |
|  | SiRe_0890 | Cmr4g7 | CRISPR-Cas system related protein, RAMP superfamily Cas7 group | 1.0327 |
|  | SiRe_0811 | - | CARF and HTH domains containing protein | 1.1224 |
|  | SiRe_0817 | - | HEPN domain containing protein | 1.0939 |
|  | SiRe_0837 | - | ATPase, predicted component of phage defense system | 1.724 |
|  | SiRe_0883 | - | ATPase, AAA+ superfamily fused to HTH and PD-(DE)xK endonuclease domains | 2.5494 |
|  | SiRe_0596 | - | HEPN domain containing protein | 1.4068 |
| Methyltransferases or | SiRe_0881 | - | SAM-dependent methyltransferase | 2.1754 |
| related | SiRe_0863 | - | SAM-dependent methyltransferase | 1.4864 |
|  | SiRe_0864 | - | SAM-dependent methyltransferase | 1.4864 |
|  | SiRe_0853 | - | SAM-dependent methyltransferase | 1.4093 |
|  | SiRe_0844 | - | SAM-dependent methyltransferase | 1.5971 |
|  | SiRe_0878 | - | SAM-dependent methyltransferase | 2.5643 |
|  | SiRe_0824 | YcaO | Ribosomal protein S12 methylthiotransferase accessory factor YcaO | 1.9989 |
| Glycosyltransferases | SiRe_0868 | - | Glycosyl transferase family 2 | 2.1262 |
|  | SiRe_0867 | RfaG | Glycosyltransferase | 2.0926 |
|  | SiRe_0876 | - | Glycosyl transferase family 2 | 2.0763 |
|  | SiRe_0874 | - | Glycosyl transferase family 2 | 1.9788 |
|  | SiRe_0854 | RfaG | Glycosyltransferase | 1.8084 |
|  | SiRe_0850 | - | Glycosyl transferase family 2 | 1.6167 |
|  | SiRe_0861 | - | Glycosyl transferase family 2 | 1.2794 |
| Other modification | SiRe_0807 | ArgE | Acetylornithine deacetylase/Succinyl-diaminopimelate desuccinylase or related deacylase | 2.5783 |
|  | SiRe_0879 | CysH | 3'-phosphoadenosine 5'-phosphosulfate sulfotransferase (PAPS reductase)/FAD synthetase | 2.3909 |
|  | SiRe_0877 | LmbE | α-N-acetylglucosaminidase or N-acetylglucosaminyl deacetylase, LmbE family | 1.9665 |
|  | SiRe_0875 | - | Arylsulfatase A or related enzyme | 1.9588 |
|  | SiRe_0841 | RfbA | dTDP-glucose pyrophosphorylase | 1.8666 |
|  | SiRe_0838 | - | TOPRIM family nucleotidyl transferase/hydrolase domain, DUF3226 family | 1.2837 |
|  | SiRe_0840 | RfbB | dTDP-D-glucose 4,6-dehydratase | 1.8007 |
|  | SiRe_0843 | RfbC | dTDP-4-dehydrorhamnose 3,5-epimerase or related enzyme | 1.6651 |
|  | SiRe_0842 | RfbD | dTDP-4-dehydrorhamnose reductase | 1.3162 |
| Transporters | SiRe_0880 | AglR | MATE family membrane protein, Rfbx family, flippase | 2.7581 |
|  | SiRe_0416 | DppC | ABC-type dipeptide/oligopeptide/nickel transport system, permease component | 2.0573 |
|  | SiRe_0417 | DppB | ABC-type dipeptide/oligopeptide/nickel transport system, permease component | 2.0339 |
|  | SiRe_0415 | DppD | ABC-type dipeptide/oligopeptide/nickel transport system, ATPase component | 1.622 |
|  | SiRe_0414 | AppF | ABC-type oligopeptide transport system, ATPase component | 1.407 |
|  | SiRe_0418 | - | Extracellular solute-binding protein with Ig-fold domain | 1.7244 |
| ROS response/Toxin- | SiRe_0857 | - | Transposase | 2.734 |
| Antitoxin/tranposon | SiRe_0806 | - | PIN domain | 2.3134 |
|  | SiRe_0453 | - | Protein distantly related to bacterial ferritins | 1.7459 |
|  | SiRe_0809 | VapC | PIN domain containing protein | 1.7129 |
|  | SiRe_0454 | {NirD} | Ferredoxin subunit of nitrite reductase or ring-hydroxylating dioxygenase | 1.6836 |
|  | SiRe_0818 | - | PIN domain containing protein | 1.4917 |
|  | SiRe_0845 | - | Transposon, IS605 OrfB family, contains RNAse H fold nuclease and Zn finger domains | 1.1862 |
| Uncharacterized | SiRe_0805 | - | Uncharacterized protein | 3.4925 |
|  | SiRe_0869 | - | Uncharacterized membrane protein | 2.0115 |
|  | SiRe_0830 | - | Uncharacterized membrane protein | 1.9019 |
|  | SiRe_0413 | - | Uncharacterized membrane protein | 1.6798 |
|  | SiRe_0127 | - | Uncharacterized membrane protein | 1.6425 |
|  | SiRe_0820 | - | Uncharacterized protein | 1.6261 |
|  | SiRe_0870 |  | ncharacterized protein | 1.5498 |
|  | SiRe_0815 | - | Uncharacterized protein | 1.4777 |
|  | SiRe_0819 | - | Uncharacterized protein | 1.0644 |
|  | SiRe_0882 |  | ncharacterized protein | 2.2051 |

**Table S7.** Observed mutations/indels in the extracted plasmids from the strain harboring wild type

SisEndoMS after induction for 60 hrs.

| **pSeSD-EndoMS*** | **Mutation**  **/Indel** | **Affected amino acids**  **or frame shift** | **Positions nt** |
| --- | --- | --- | --- |
| Plasmid 1 | C-T | **C**CC: Proline (P)→**T**CC: Serine (S) | 409 (a.a.137) |
| Plasmid 2 | Insertion (A) | Insertion (A) | 499 (after a.a.166) |
| Plasmid 3 | C-T | **C**CC: Proline (P)→**T**CC: Serine (S) | 409 (a.a.137) |
| Plasmid 4 | Insertion (G) | Insertion (G) | 580 (after a.a. 193) |
| Plasmid 5 | Insertion (A) | Insertion (A) | 498 (after a.a.166) |
| Plasmid 6 | C-T | **C**CC: Proline (P)→**T**CC: Serine (S) | 409 (a.a. 137) |

*pSeSD-EndoMS plasmid extracted from six independently grown EndoMS overexpression cultures.

Nt, nucleotide; a.a., amino acid.
