# Supplementary material for: Functional Analysis of the NucS/EndoMS of the Hyperthermophilic Archaeon Sulfolobus islandicus REY15A
Source: Front Microbiol. 2020 Dec 1;11:607431. doi: 10.3389/fmicb.2020.607431 (PMC7736090; doi:10.3389/fmicb.2020.607431)
Supplement: Supplementary file 2 [file Presentation_1.pptx]

## Slide 1
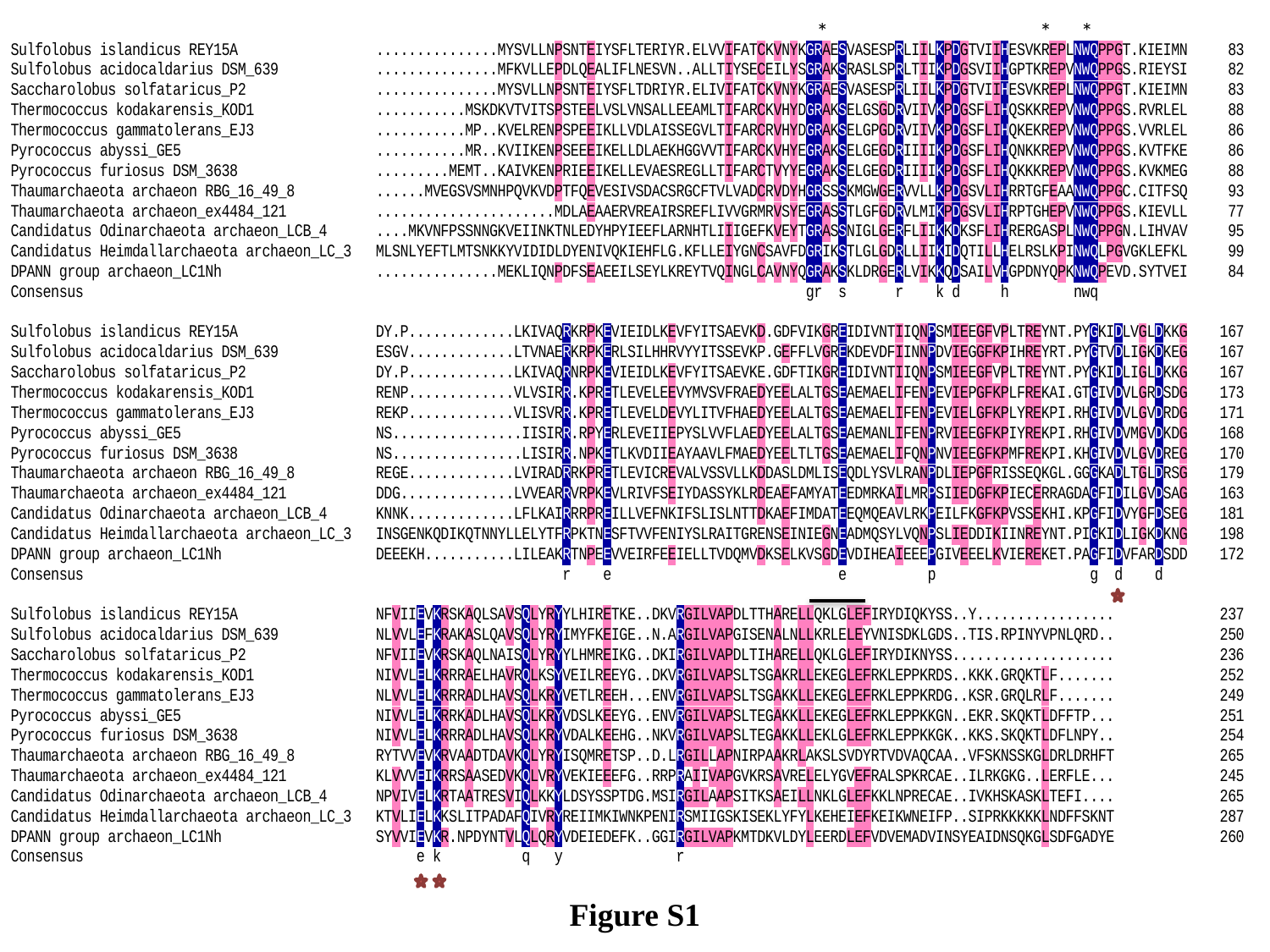

*
*
*
Figure S1

## Slide 2
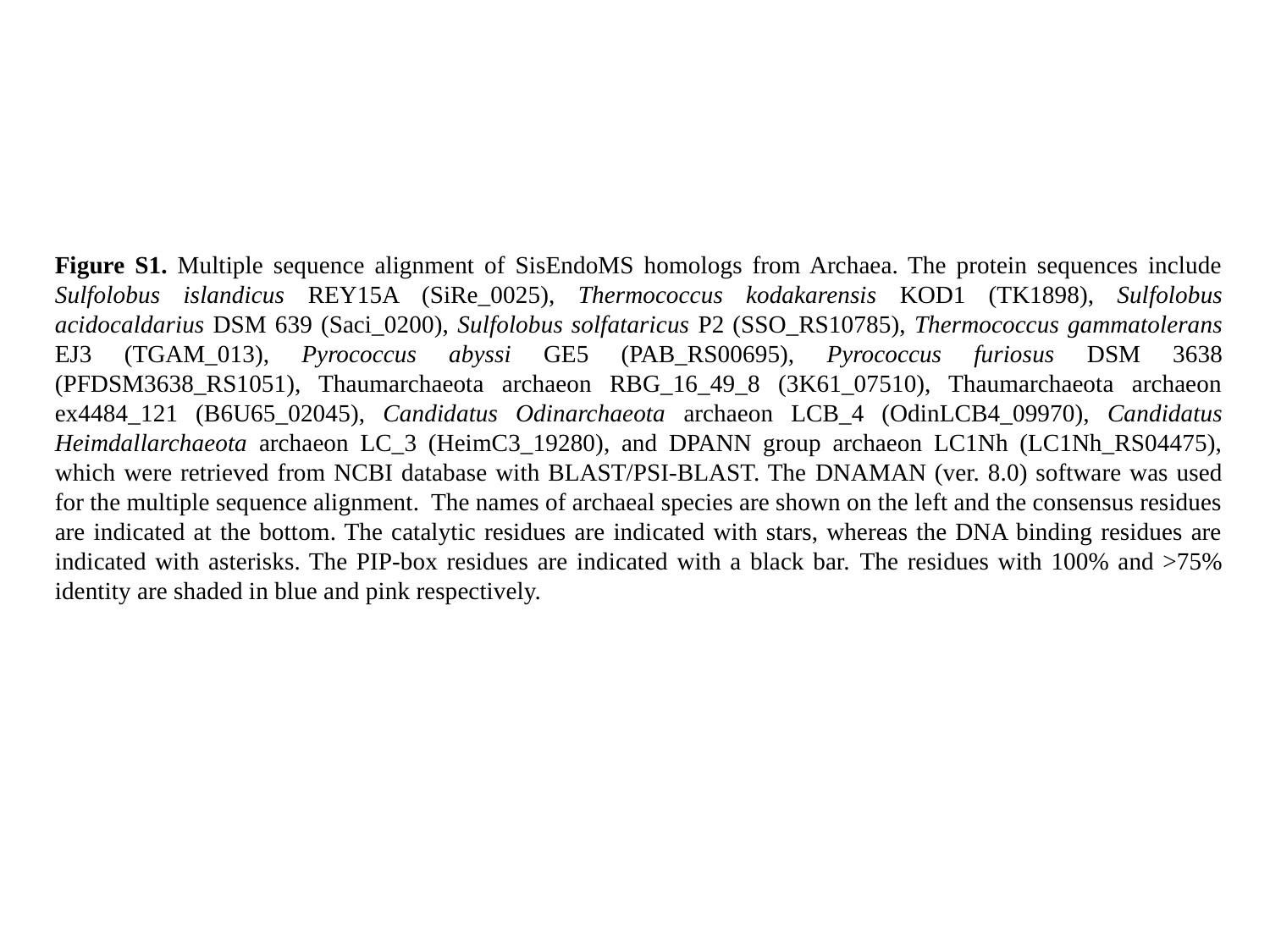

Figure S1. Multiple sequence alignment of SisEndoMS homologs from Archaea. The protein sequences include Sulfolobus islandicus REY15A (SiRe_0025), Thermococcus kodakarensis KOD1 (TK1898), Sulfolobus acidocaldarius DSM 639 (Saci_0200), Sulfolobus solfataricus P2 (SSO_RS10785), Thermococcus gammatolerans EJ3 (TGAM_013), Pyrococcus abyssi GE5 (PAB_RS00695), Pyrococcus furiosus DSM 3638 (PFDSM3638_RS1051), Thaumarchaeota archaeon RBG_16_49_8 (3K61_07510), Thaumarchaeota archaeon ex4484_121 (B6U65_02045), Candidatus Odinarchaeota archaeon LCB_4 (OdinLCB4_09970), Candidatus Heimdallarchaeota archaeon LC_3 (HeimC3_19280), and DPANN group archaeon LC1Nh (LC1Nh_RS04475), which were retrieved from NCBI database with BLAST/PSI-BLAST. The DNAMAN (ver. 8.0) software was used for the multiple sequence alignment. The names of archaeal species are shown on the left and the consensus residues are indicated at the bottom. The catalytic residues are indicated with stars, whereas the DNA binding residues are indicated with asterisks. The PIP-box residues are indicated with a black bar. The residues with 100% and >75% identity are shaded in blue and pink respectively.

## Slide 3
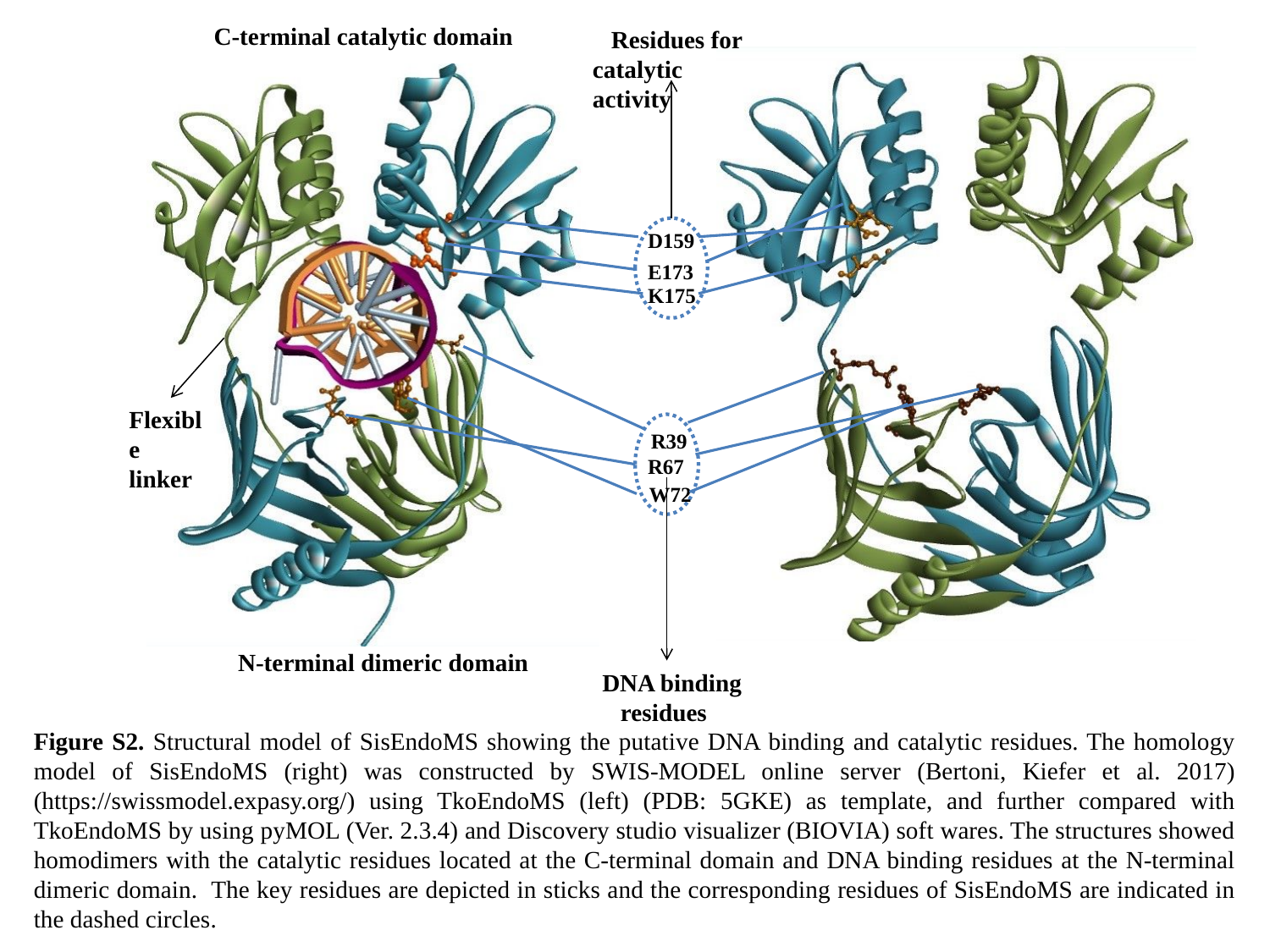

C-terminal catalytic domain
 Residues for catalytic activity
D159
E173
K175
Flexible
linker
R39
R67
W72
N-terminal dimeric domain
 DNA binding
 residues
Figure S2. Structural model of SisEndoMS showing the putative DNA binding and catalytic residues. The homology model of SisEndoMS (right) was constructed by SWIS-MODEL online server (Bertoni, Kiefer et al. 2017) (https://swissmodel.expasy.org/) using TkoEndoMS (left) (PDB: 5GKE) as template, and further compared with TkoEndoMS by using pyMOL (Ver. 2.3.4) and Discovery studio visualizer (BIOVIA) soft wares. The structures showed homodimers with the catalytic residues located at the C-terminal domain and DNA binding residues at the N-terminal dimeric domain. The key residues are depicted in sticks and the corresponding residues of SisEndoMS are indicated in the dashed circles.

## Slide 4
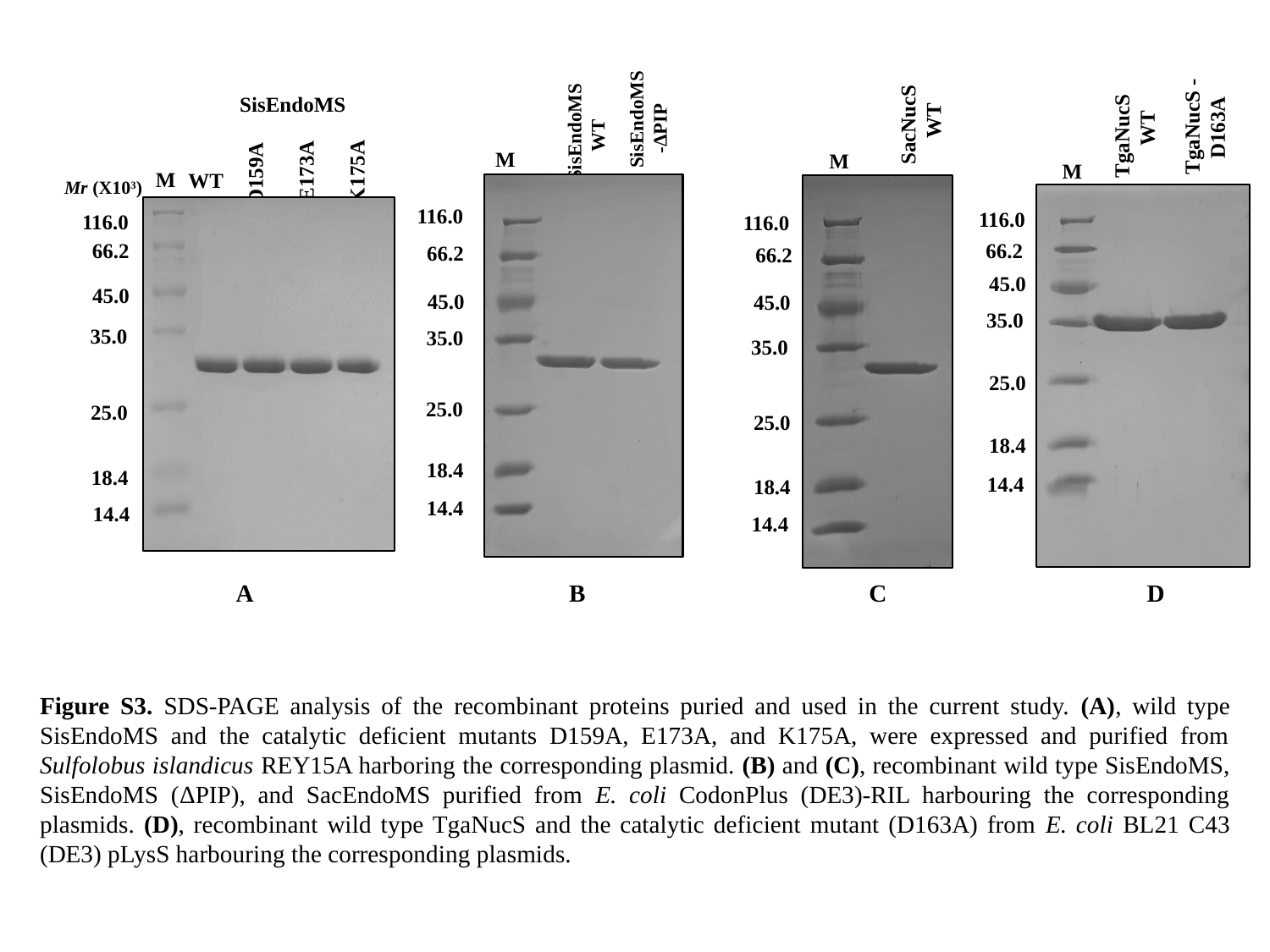

SacNucS
 WT
M
116.0
66.2
45.0
35.0
25.0
18.4
14.4
SisEndoMS
 WT
M
116.0
66.2
45.0
35.0
25.0
18.4
14.4
SisEndoMS
E173A
M
K175A
WT
D159A
116.0
66.2
45.0
35.0
25.0
18.4
14.4
Mr (X103)
SisEndoMS
 -ΔPIP
TgaNucS -
 D163A
TgaNucS
 WT
M
116.0
66.2
45.0
35.0
25.0
18.4
14.4
A
B
C
D
Figure S3. SDS-PAGE analysis of the recombinant proteins puried and used in the current study. (A), wild type SisEndoMS and the catalytic deficient mutants D159A, E173A, and K175A, were expressed and purified from Sulfolobus islandicus REY15A harboring the corresponding plasmid. (B) and (C), recombinant wild type SisEndoMS, SisEndoMS (ΔPIP), and SacEndoMS purified from E. coli CodonPlus (DE3)-RIL harbouring the corresponding plasmids. (D), recombinant wild type TgaNucS and the catalytic deficient mutant (D163A) from E. coli BL21 C43 (DE3) pLysS harbouring the corresponding plasmids.

## Slide 5
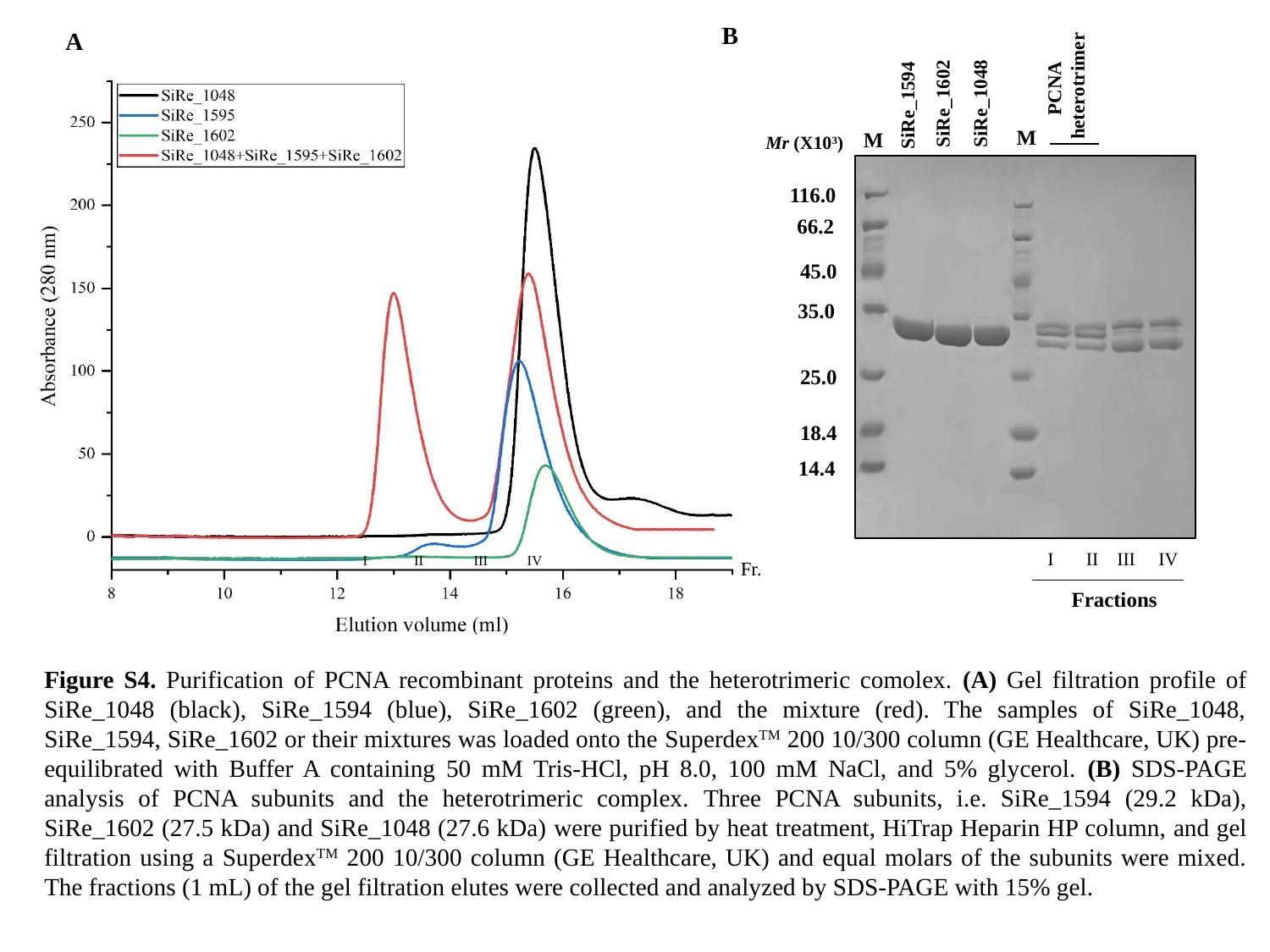

B
 PCNA
heterotrimer
SiRe_1602
SiRe_1048
SiRe_1594
M
M
Fractions
Mr (X103)
116.0
66.2
45.0
35.0
25.0
18.4
14.4
A
I II III IV
I II III IV
Fr.
Figure S4. Purification of PCNA recombinant proteins and the heterotrimeric comolex. (A) Gel filtration profile of SiRe_1048 (black), SiRe_1594 (blue), SiRe_1602 (green), and the mixture (red). The samples of SiRe_1048, SiRe_1594, SiRe_1602 or their mixtures was loaded onto the SuperdexTM 200 10/300 column (GE Healthcare, UK) pre-equilibrated with Buffer A containing 50 mM Tris-HCl, pH 8.0, 100 mM NaCl, and 5% glycerol. (B) SDS-PAGE analysis of PCNA subunits and the heterotrimeric complex. Three PCNA subunits, i.e. SiRe_1594 (29.2 kDa), SiRe_1602 (27.5 kDa) and SiRe_1048 (27.6 kDa) were purified by heat treatment, HiTrap Heparin HP column, and gel filtration using a SuperdexTM 200 10/300 column (GE Healthcare, UK) and equal molars of the subunits were mixed. The fractions (1 mL) of the gel filtration elutes were collected and analyzed by SDS-PAGE with 15% gel.

## Slide 6
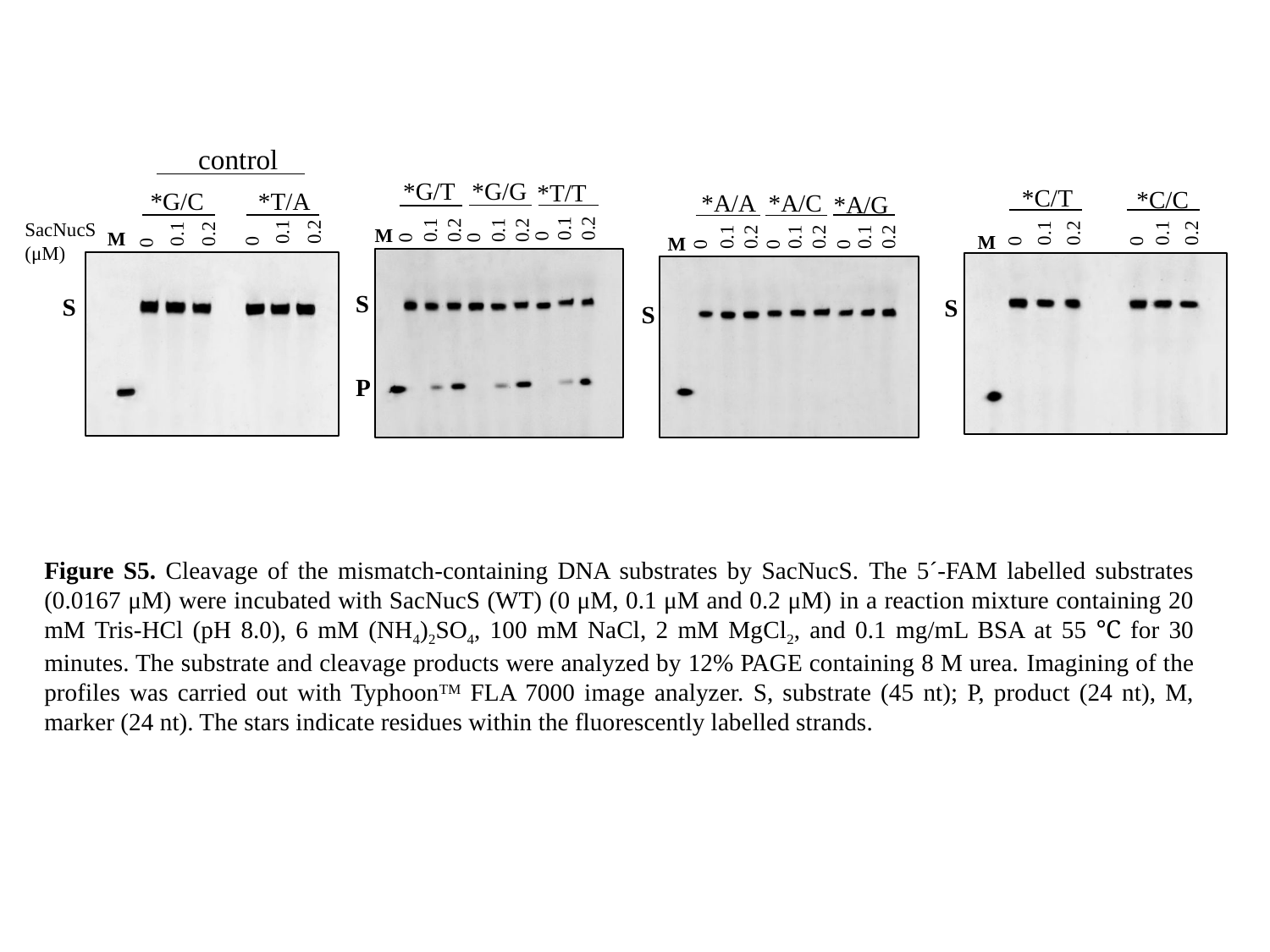

control
*G/C
*T/A
0.1
0.2
0
0.1
0.2
0
SacNucS(μM)
M
S
*G/T
*G/G
*T/T
0.1
0.2
0
0.1
0.2
0
0.1
0.2
0
M
S
P
*C/T
*C/C
0.2
0.1
0
0.2
0.1
0
M
S
*A/C
*A/A
*A/G
0.1
0.2
0
0.1
0.2
0
0.1
0.2
0
M
S
Figure S5. Cleavage of the mismatch-containing DNA substrates by SacNucS. The 5´-FAM labelled substrates (0.0167 μM) were incubated with SacNucS (WT) (0 μM, 0.1 μM and 0.2 μM) in a reaction mixture containing 20 mM Tris-HCl (pH 8.0), 6 mM (NH4)2SO4, 100 mM NaCl, 2 mM MgCl2, and 0.1 mg/mL BSA at 55 ℃ for 30 minutes. The substrate and cleavage products were analyzed by 12% PAGE containing 8 M urea. Imagining of the profiles was carried out with TyphoonTM FLA 7000 image analyzer. S, substrate (45 nt); P, product (24 nt), M, marker (24 nt). The stars indicate residues within the fluorescently labelled strands.

## Slide 7
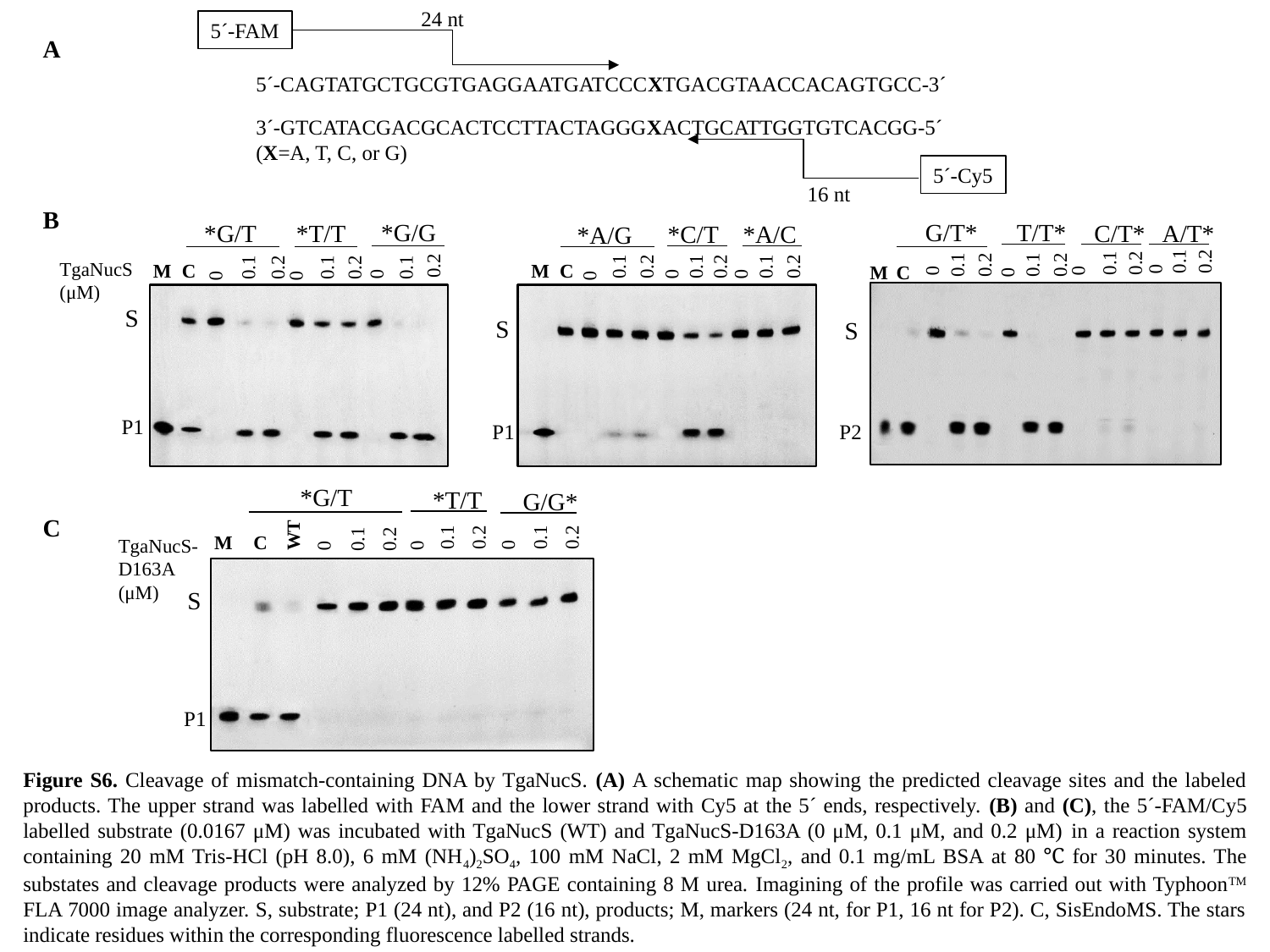

24 nt
5´-FAM
5´-CAGTATGCTGCGTGAGGAATGATCCCXTGACGTAACCACAGTGCC-3´
3´-GTCATACGACGCACTCCTTACTAGGGXACTGCATTGGTGTCACGG-5´
(X=A, T, C, or G)
5´-Cy5
16 nt
A
B
G/T*
T/T*
C/T*
A/T*
0.1
0.2
0
0.1
0.2
0
0.1
0.2
0
0.1
0.2
0
M
C
S
P2
*G/G
*G/T
*T/T
0.2
0.1
0
0.1
0.2
0
0.1
0.2
0
TgaNucS
(μM)
M
C
S
P1
*C/T
*A/C
*A/G
0.1
0.2
0
0.1
0.2
0
0.1
0.2
0
M
C
S
P1
*G/T
*T/T
G/G*
0.2
0.1
0
0.2
0.1
0
0.2
0.1
0
WT
C
M
TgaNucS-
D163A
(μM)
S
P1
C
Figure S6. Cleavage of mismatch-containing DNA by TgaNucS. (A) A schematic map showing the predicted cleavage sites and the labeled products. The upper strand was labelled with FAM and the lower strand with Cy5 at the 5´ ends, respectively. (B) and (C), the 5´-FAM/Cy5 labelled substrate (0.0167 μM) was incubated with TgaNucS (WT) and TgaNucS-D163A (0 μM, 0.1 μM, and 0.2 μM) in a reaction system containing 20 mM Tris-HCl (pH 8.0), 6 mM (NH4)2SO4, 100 mM NaCl, 2 mM MgCl2, and 0.1 mg/mL BSA at 80 ℃ for 30 minutes. The substates and cleavage products were analyzed by 12% PAGE containing 8 M urea. Imagining of the profile was carried out with TyphoonTM FLA 7000 image analyzer. S, substrate; P1 (24 nt), and P2 (16 nt), products; M, markers (24 nt, for P1, 16 nt for P2). C, SisEndoMS. The stars indicate residues within the corresponding fluorescence labelled strands.

## Slide 8
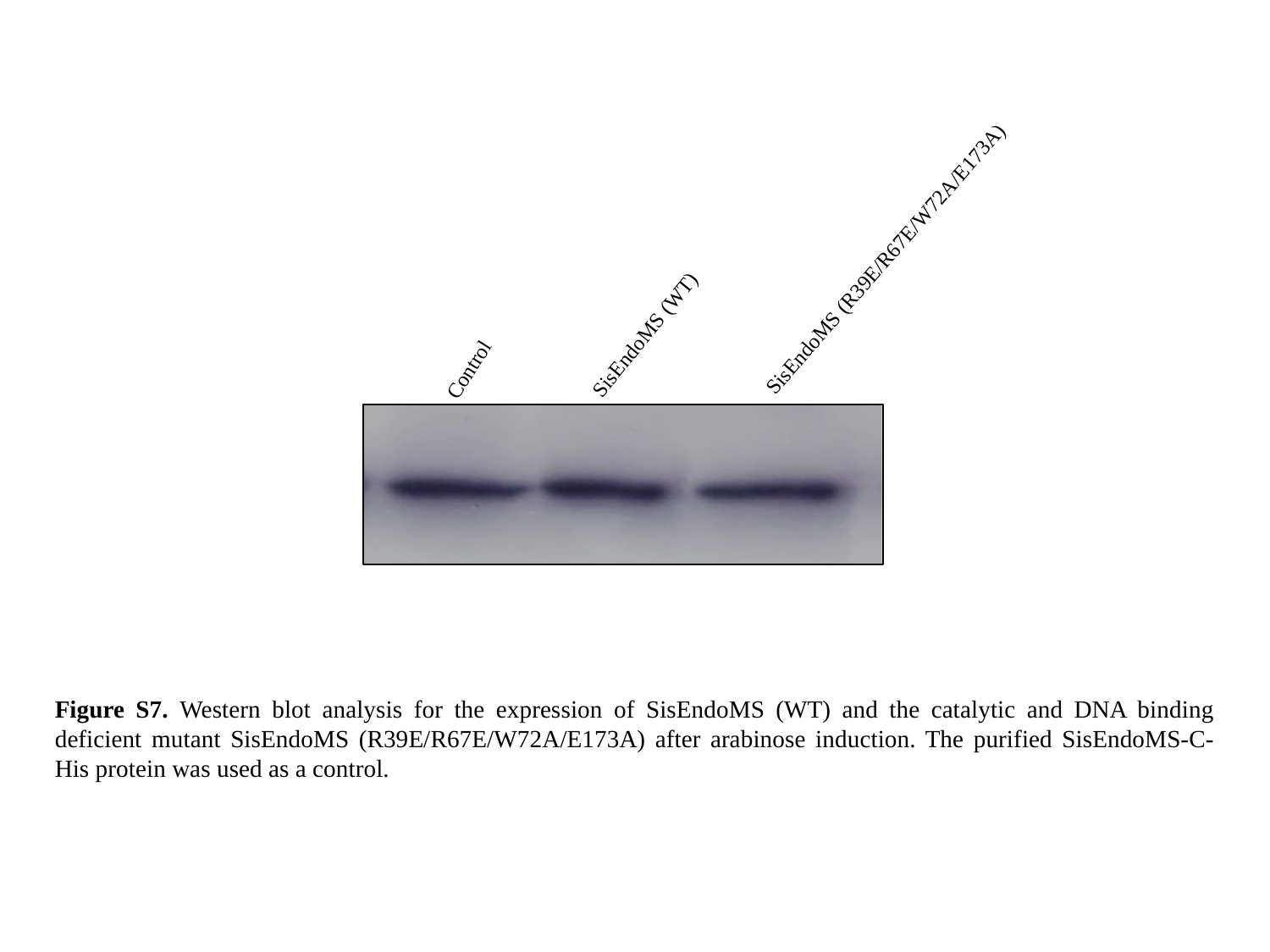

SisEndoMS (R39E/R67E/W72A/E173A)
SisEndoMS (WT)
Control
Figure S7. Western blot analysis for the expression of SisEndoMS (WT) and the catalytic and DNA binding deficient mutant SisEndoMS (R39E/R67E/W72A/E173A) after arabinose induction. The purified SisEndoMS-C-His protein was used as a control.

## Slide 9
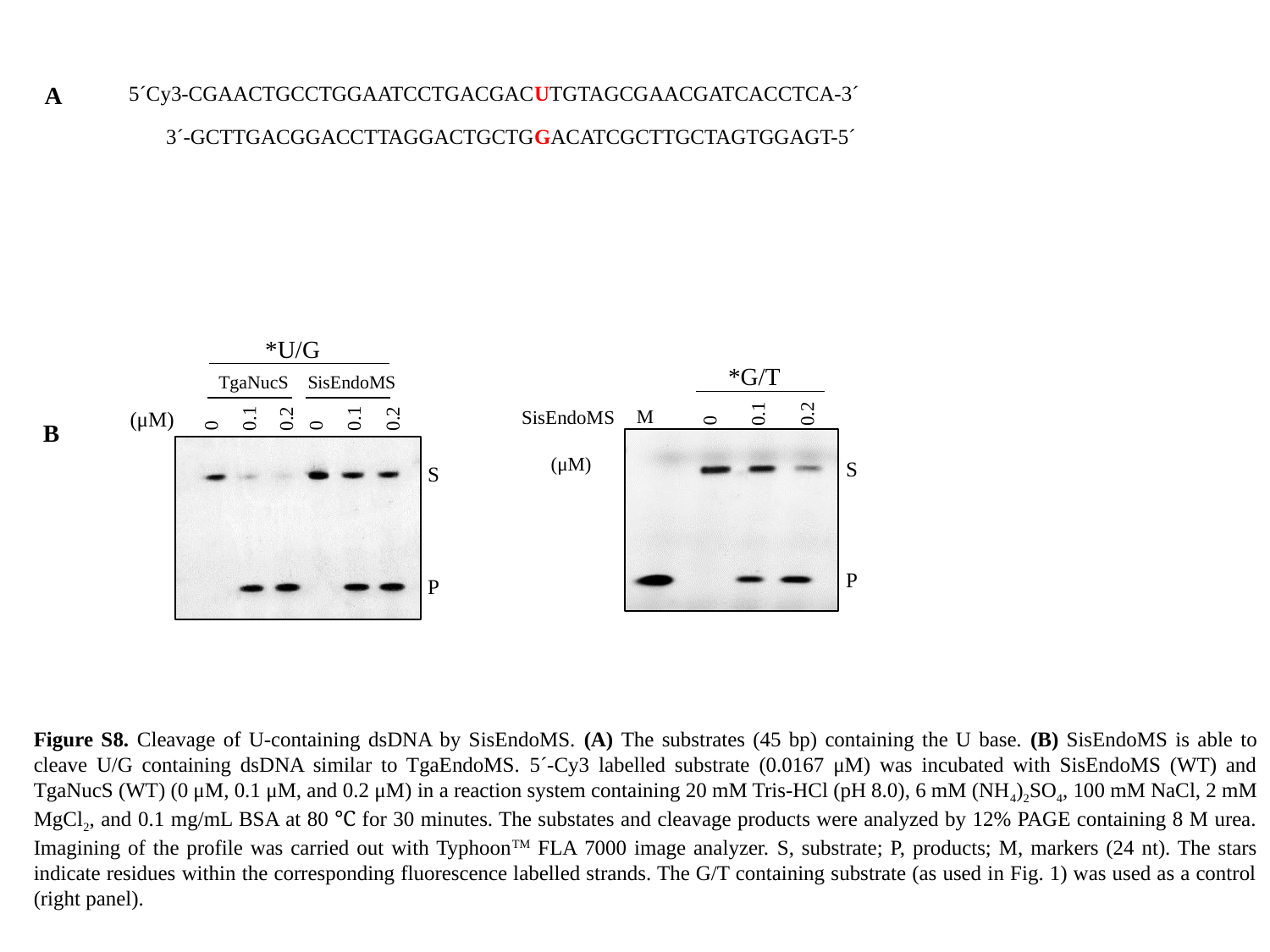

A
5´Cy3-CGAACTGCCTGGAATCCTGACGACUTGTAGCGAACGATCACCTCA-3´
 3´-GCTTGACGGACCTTAGGACTGCTGGACATCGCTTGCTAGTGGAGT-5´
*U/G
TgaNucS
SisEndoMS
0.2
0.1
0
0.2
0.1
0
(μM)
S
P
*G/T
0.2
0.1
0
M
SisEndoMS
 (μM)
S
P
B
Figure S8. Cleavage of U-containing dsDNA by SisEndoMS. (A) The substrates (45 bp) containing the U base. (B) SisEndoMS is able to cleave U/G containing dsDNA similar to TgaEndoMS. 5´-Cy3 labelled substrate (0.0167 μM) was incubated with SisEndoMS (WT) and TgaNucS (WT) (0 μM, 0.1 μM, and 0.2 μM) in a reaction system containing 20 mM Tris-HCl (pH 8.0), 6 mM (NH4)2SO4, 100 mM NaCl, 2 mM MgCl2, and 0.1 mg/mL BSA at 80 ℃ for 30 minutes. The substates and cleavage products were analyzed by 12% PAGE containing 8 M urea. Imagining of the profile was carried out with TyphoonTM FLA 7000 image analyzer. S, substrate; P, products; M, markers (24 nt). The stars indicate residues within the corresponding fluorescence labelled strands. The G/T containing substrate (as used in Fig. 1) was used as a control (right panel).

## Slide 10
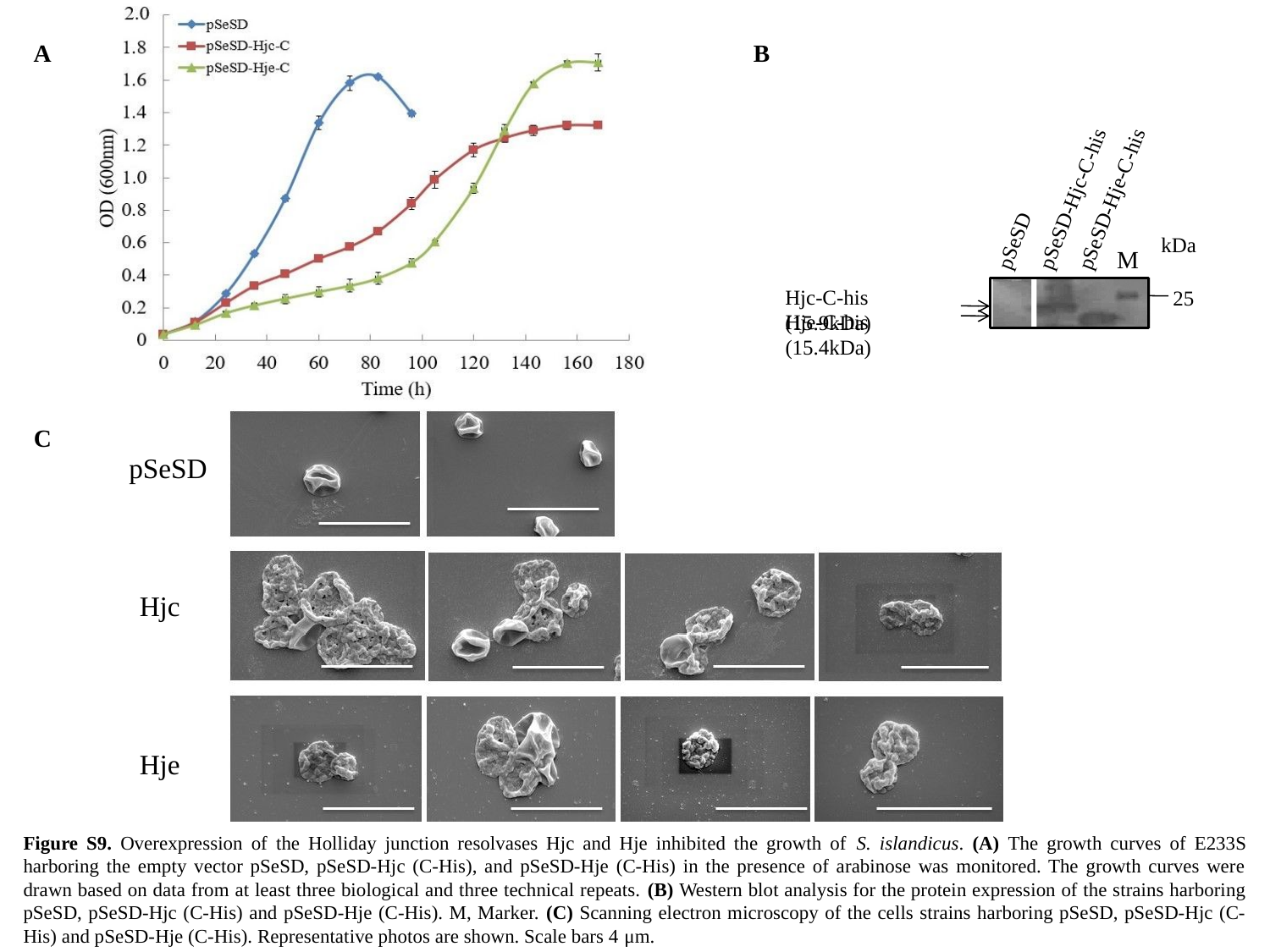

A
B
pSeSD-Hjc-C-his
pSeSD-Hje-C-his
pSeSD
kDa
M
Hjc-C-his (15.9kDa)
25
Hje-C-his (15.4kDa)
C
pSeSD
Hjc
Hje
# Figure S9. Overexpression of the Holliday junction resolvases Hjc and Hje inhibited the growth of S. islandicus. (A) The growth curves of E233S harboring the empty vector pSeSD, pSeSD-Hjc (C-His), and pSeSD-Hje (C-His) in the presence of arabinose was monitored. The growth curves were drawn based on data from at least three biological and three technical repeats. (B) Western blot analysis for the protein expression of the strains harboring pSeSD, pSeSD-Hjc (C-His) and pSeSD-Hje (C-His). M, Marker. (C) Scanning electron microscopy of the cells strains harboring pSeSD, pSeSD-Hjc (C-His) and pSeSD-Hje (C-His). Representative photos are shown. Scale bars 4 μm.
